# FUS Mislocalization and Vulnerability to DNA Damage in ALS Patients Derived hiPSCs and Aging Motoneurons

**DOI:** 10.3389/fncel.2016.00290

**Published:** 2016-12-26

**Authors:** Julia Higelin, Maria Demestre, Stefan Putz, Jan P. Delling, Christian Jacob, Anne-Kathrin Lutz, Julia Bausinger, Anne-Kathrin Huber, Moritz Klingenstein, Gotthold Barbi, Günter Speit, Annemarie Huebers, Jochen H. Weishaupt, Andreas Hermann, Stefan Liebau, Albert C. Ludolph, Tobias M. Boeckers

**Affiliations:** ^1^Institute for Anatomy and Cell Biology, Ulm UniversityUlm, Germany; ^2^Department of Neurology, Ulm UniversityUlm, Germany; ^3^Institute for Human Genetics, Ulm UniversityUlm, Germany; ^4^Institute of Neuroanatomy, Eberhard Karls University of TübingenTübingen, Germany; ^5^Department of Neurology, Technische Universität DresdenDresden, Germany; ^6^German Center for Neurodegenerative DiseasesDresden, Germany; ^7^Center for Regenerative Therapies Dresden, Technische Universität DresdenDresden, Germany

**Keywords:** hiPSC, FUS, ALS, motoneuron, neurodegeneration, DNA damage, FTLD

## Abstract

Mutations within the *FUS* gene (Fused in Sarcoma) are known to cause Amyotrophic Lateral Sclerosis (ALS), a neurodegenerative disease affecting upper and lower motoneurons. The *FUS* gene codes for a multifunctional RNA/DNA-binding protein that is primarily localized in the nucleus and is involved in cellular processes such as splicing, translation, mRNA transport and DNA damage response. In this study, we analyzed pathophysiological alterations associated with ALS related FUS mutations (mFUS) in human induced pluripotent stem cells (hiPSCs) and hiPSC derived motoneurons. To that end, we compared cells carrying a mild or severe mFUS in physiological- and/or stress conditions as well as after induced DNA damage. Following hyperosmolar stress or irradiation, mFUS hiPS cells recruited significantly more cytoplasmatic FUS into stress granules accompanied by impaired DNA-damage repair. In motoneurons wild-type FUS was localized in the nucleus but also deposited as small punctae within neurites. In motoneurons expressing mFUS the protein was additionally detected in the cytoplasm and a significantly increased number of large, densely packed FUS positive stress granules were seen along neurites. The amount of FUS mislocalization correlated positively with both the onset of the human disease (the earlier the onset the higher the FUS mislocalization) and the maturation status of the motoneurons. Moreover, even in non-stressed post-mitotic mFUS motoneurons clear signs of DNA-damage could be detected. In summary, we found that the susceptibility to cell stress was higher in mFUS hiPSCs and hiPSC derived motoneurons than in controls and the degree of FUS mislocalization correlated well with the clinical severity of the underlying ALS related mFUS. The accumulation of DNA damage and the cellular response to DNA damage stressors was more pronounced in post-mitotic mFUS motoneurons than in dividing hiPSCs suggesting that mFUS motoneurons accumulate foci of DNA damage, which in turn might be directly linked to neurodegeneration.

## Introduction

Amyotrophic Lateral Sclerosis (ALS) is a fatal neurodegenerative disorder characterized by the selective demise of upper and lower motoneurons (MNs). In most patients, the disease is sporadic and the pathogenesis is unknown. The identification of genetic causes [i.e., mutations in of super oxide dismutase-1 (SOD-1)] gave another scope into understanding familial ALS (fALS). In 2006, the RNA binding protein TAR DNA-binding protein 43 (TDP-43) encoded by the *TARDBP* gene was identified as a major component of ubiquitinated aggregates in ALS and frontotemporal lobar degeneration (FTLD) (Arai et al., 2006; Neumann et al., 2006). The identification of TDP-43 as an important protein in ALS-pathogenesis directly triggered the discovery of further ALS and FTLD related mutations in the RNA/DNA-binding protein FUS (Kwiatkowski et al., 2009; Vance et al., 2009; Blair et al., 2010). FUS is predominantly found in nuclei (Anderson and Kedersha, 2009) but is also able to shuttle between the nucleus and the cytoplasm (Dormann and Haass, 2011). FUS seems to be an important factor for the nuclear export of messenger RNA (mRNA) and the dendritic transport of mRNA for local translation in neurons (Fujii and Takumi, 2005; Fujii et al., 2005). Furthermore, FUS-positive granules co-localizing with synaptic markers are also present along dendrites of mouse neurons and also in the human brain, suggesting an additional role at synaptic sites (Belly et al., 2010; Aoki et al., 2012; Schoen et al., 2016). In this respect, it has been described that upon synaptic mGluR5 activation FUS is translocated to dendritic spines. FUS deficient mice display disturbed spine maturation and excessive dendritic branching (Fujii and Takumi, 2005; Fujii et al., 2005). Similarly, transgenic mice expressing the FUS mutation R521C have transcription and splicing defects in genes that regulate dendrite outgrowth and synaptic function (Qiu et al., 2014).

In affected patients carrying FUS mutations, FUS is partially or totally excluded from the nucleus and forms cytoplasmic inclusions in neurons (and in glial cells) of the brain and spinal cord (Neumann et al., 2009; Vance et al., 2009; Dormann et al., 2010). In some cells, intra-nuclear inclusions have been described (Neumann et al., 2009; Woulfe et al., 2010). Interestingly, FUS-ALS-linked mutations are mainly clustered at the C-terminal region of the protein, which contains the nuclear localization signal (NLS). Therefore, deletions or mutations in the NLS could explain the increased cytoplasmic distribution of the FUS protein. Increasing levels of cytoplasmic FUS are associated with a more aggressive course of the disease, meaning that mutations that induce a strong nuclear import defect are usually associated with an early disease onset and fast disease progression (Bosco et al., 2010; DeJesus-Hernandez et al., 2010; Dormann et al., 2010).

Up to now, the exact pathomechanism induced by mutated FUS (mFUS) in ALS still remains unclear, but there is evidence that under physiological conditions FUS is involved in DNA damage responses (DDR) as well as RNA processing and transcription. In this respect, it was shown that FUS is a component of DDR machinery since wild type (WT)-FUS is recruited to DNA damage foci in neurons and interacts with histone deacyclase-1 (HDAC1), a chromatin modifying enzyme involved in DDR signaling and DNA repair. Mutations in FUS, however, presented a weaker HDAC1-interaction leading to an impaired DDR in neurons. In addition, neuropathological examination revealed increased DNA damage in cortical and spinal neurons from fALS patients expressing mFUS variants (Wang et al., 2013). Along these lines, transgenic mice expressing human variant FUS-R521C, presented enhanced DNA damage in cortical and spinal motoneurons and exhibited DNA repair defects in the 5′ non coding exons of the brain derived neurotrophic factor gene (Qiu et al., 2014). Moreover, several studies have demonstrated that ALS related FUS mutations are directly associated with the formation of cytoplasmic stress granules (SGs) under stress conditions such as heat shock and hyperosmolarity (Anderson and Kedersha, 2009). In fact, pathological inclusions found in brains and spinal cords from fALS-FUS patients are positive for SG markers (Bentmann et al., 2012). After hyperosmolar stress, endogenous FUS is redistributed to the cytoplasm and localized to SGs as demonstrated by Sama et al. (2014b) in HeLA cells. However, compared to mFUS that sequesters very strongly into SGs, this process was found to be less efficient (Bosco et al., 2010; Dormann et al., 2010; Ito et al., 2011; Bentmann et al., 2012).

The cellular and molecular analysis of ALS pathogenesis has been impeded by the absence of suitable cell model systems, in which mutated proteins are usually overexpressed. Post mortem tissues are also difficult to obtain and mouse models have been useful but they do not reflect the complete pathology seen in humans. In this study, we took advantage of the generation of human induced pluripotent stem cells (hiPSCs) by reprogramming somatic cells (Aasen et al., 2008; Patel and Yang, 2010) and derived into various lineages, including spinal MNs (Stockmann et al., 2013). More importantly, patient-derived iPSCs lines expressing endogenous mFUS were analyzed. This provides the possibility to identify the pathophysiological characteristics of different mutations based directly on patient specific settings.

We addressed whether the above described pathophysiological phenotypes associated with mFUS, i.e., FUS mislocalization, SG formation and enhanced DNA damage are present in hiPSCs derived from three different fALS-FUS patients under physiological and stress conditions as well as after induced DNA damage. To this end hiPS cell lines were generated from fALS-FUS patients harboring three different endogenous mutations (all heterozygous) in the FUS C-terminus.

Only a few studies using hiPS cell lines expressing different mFUS variants are published up to now showing a cytoplasmatic distribution of FUS in mFUS hiPSC derived spinal MN (Lenzi et al., 2015; Liu et al., 2015; Ichiyanagi et al., 2016; Naujock et al., 2016) and an enhanced recruitment of FUS into SGs after the induction of cellular stress. Similarly, in hiPSC-derived cortical neurons expressing different mFUS variants (a mild mutation R521C and an aggressive mutation R495QfsX527), cytoplasmic FUS inclusions were spontaneously formed and were dependent on both, the severity of the mutation and neuronal age. Furthermore, the severity of the FUS mutation determined the cell vulnerability to oxidative stress (Japtok et al., 2015). In addition to the two previously described mutations, we generated and differentiated an additional hiPSC line from a juvenile ALS-patient with a severe *de novo* FUS mutation Asp502Thrfs^∗^27 (Hübers et al., 2015). Undifferentiated cells were not only exposed to hyperosmolar stress (Lenzi et al., 2015) but also to DNA damage induction. We found that hyperosmolar stress as well as DNA damage induced preferentially recruitment of FUS into SGs in cells expressing mFUS and this correlated well with the severity of the underlying mutation. More interestingly, iPSCs were unable to repair the DNA properly. In spinal MNs the extra-nuclear localization of FUS was directly correlated with the stage of neuronal aging and the onset of the disease. Young MNs harboring the most aggressive mutation presented FUS delocalization and spontaneous DNA-damage accumulation. Moreover, in control cells FUS was detectable in small punctae along neurites (Belly et al., 2010; Aoki et al., 2012; Schoen et al., 2016). In mutant MN, however, an increased number of large, densely packed FUS positive SG were formed spontaneously along the neurites.

## Materials and Methods

### Cultivation of Human Keratinocytes and Mouse Embryonic Fibroblasts

Generation of iPSC lines from hair keratinocytes as well as the cultivation of CD-1 mouse embryonic fibroblasts (MEFs) (day E14.5) (Stemcell Technologies, Vancouver; CA, USA) was performed as previously described (Takahashi and Yamanaka, 2006; Aasen et al., 2008).

### Generation of Lentiviruses

Viral particles were produced according to published protocols. For more detailed information, see Linta et al. (2012) and Stockmann et al. (2013).

### Generation and Cultivation of Human iPSCs

Human keratinocytes were reprogrammed as previously described (Stockmann et al., 2013) using a lentiviral polycystronic STEMCCA cassette encoding *octamer-binding transcription factor 4* (*OCT4*), *sex determining region Y-box 2* (*SOX2*), *Kruppel-like factor 4* **(***KLF4*), and *c-MYC* (Somers et al., 2012). Briefly, keratinocytes (75% confluence) were treated with 5 × 10^5^ proviral genome copies in EpiLife medium (Invitrogen, Carlsbad, CA, USA) supplemented with 8 μg/ml polybrene (Sigma-Aldrich, St. Louis, MO, USA) on two subsequent days. Afterwards, keratinocytes were detached with TrypLE Express (Invitrogen, Carlsbad, CA, USA) for 10 min at 37°C and transferred to previously irradiated (30Gy) rat embryonic fibroblasts feeder cells. Keratinocytes were cultured in hiPSC medium consisting of knockout/DMEM supplemented with 20% knockout serum replacement, 2 mM GlutaMAX, 100 mM non-essential amino acids (all from Life technologies), 1% Antibiotic–Antimycotic, 100 mM β-mercaptoethanol (Millipore, USA), 50 mg/ml vitamin C, and 10 ng/ml fibroblast growth factor (FGF-2) (both from PeproTech, USA) in a 5% O_2_ incubator. Medium was changed daily. After 3–5 days small colonies appeared with typical hiPSCs morphology. Around 14 days later, hiPSC colonies had the appropriate size for mechanical passaging and were transferred onto irradiated MEFs (Stem Cell Technologies, France) and further cultivated with hiPSC medium. After one passage hiPSC colonies were mechanically picked and transferred to feeder free plates and maintained with mTReSR1 medium (Stem Cell Technologies, France). For splitting, hiPSCs colonies were incubated with dispase (StemCell Technologies) for 5–7 min at 37°C and subsequently detached using a cell scraper. For more detailed methods see Stockmann et al. (2013).

The reprogramming of the human fibroblasts was performed essentially as described by Reinhardt et al. (2013) and Lojewski et al. (2014) using pMX-based retroviral vectors encoding human Yamanaka factors (Reinhardt et al., 2013; Lojewski et al., 2014). For infection, up to 50 000 fibroblasts per well of a 0.1% gelatin-coated 6-well-plate were infected three times with pMX vectors in combination with 6 μg/ml protamine sulfate (Sigma-Aldrich) and 5 ng/ml FGF-2 (Peprotech). Infected fibroblasts were plated onto mitomycin C (MMC, Tocris) inactivated CF-1-MEFs. The next day media was exchanged to ES medium containing 78% Knock-out DMEM, 20% Knock-out serum replacement, 1% non-essential amino acids, 1% penicillin/streptomycin/glutamine and 50 μM β-Mercaptoethanol (all from Invitrogen) supplemented with 5 ng/ml FGF-2 and 1 mM valproic acid (Sigma Aldrich). Media was changed every day to the same conditions. Seven days after viral infection hiPSC-like cells appeared and were cultured for additional 7 days. At day 14 post-infection, the cells were manually picked and plated on CF-1 feeder cells in ES medium supplemented with 5 ng/ml FGF-2. By using 1 mg/ml collagenase type IV (Invitrogen) constant colonies were passaged on CF-1 feeder cells (Globalstem, Gaithersburg, MD, USA) treated with MMC. Addition of 10 μM Rock-inhibitor Y-27632 for the first 48 h improved survival of hiPSCs. Medium was changed daily and contained FGF-2. The cultivation of generated hiPSCs at later passages was performed essentially as described (Stockmann et al., 2013) under feeder- and serum-free conditions.

### Characterization of Pluripotency and *In vitro* Differentiation of EBs

The pluripotency tests and the germ-layer-specific detection was performed as previously described (Linta et al., 2012; Stockmann et al., 2013) or done according to the manufacturer’s protocol using the StemLite Pluripotency Kit (Cell Signaling, Danvers, MA, USA). For *in vitro* differentiation, hiPSC colonies were mechanically lifted by dispase (Stemcell Technologies) digested and transferred in T75 low-attachment flasks (Corning, Corning, NY, USA). Embryoid body (EB) formation was performed in suspension in knockout DMEM supplemented with 2 mM GlutaMAX, 20% Knockout Serum Replacement (Invitrogen, Carlsbad, CA, USA), 1% Antibiotic-Antimycotic (Invitrogen), 100 μM nonessential amino acids (Invitrogen), and 100 μM ß-mercaptoethanol (Invitrogen) for 10 days. Afterwards, EBs were plated on 35 mm dishes (Ibidi, Munich, BY, Germany) and kept in culture for up to 14 days. Differentiated EBs were stained 1:1000 chicken anti-Tubulin beta-III (Millipore, Billerica, MA, USA); 1:150 mouse anti-Actinin (Sigma Aldrich) and 1:100 goat anti-AFP (Santa Cruz, CA, USA). Alexa fluor secondary antibodies were used from Invitrogen.

### Karyotyping

Karyograms of all generated iPSC lines were analyzed to exclude chromosomal aberrations after reprograming. Chromosome preparation of hiPS cells was performed according to standard procedures (Linta et al., 2012). Briefly, two confluent wells of a six-well-plate were prepared. 1.5 M Colchicine (20 mg/l, Eurobio, Courtaboeuf, France) was added for 2 h to conserve metaphases. Treated cells were detached with TrypLE for 4 min at 37°C. Cultures represent normal male/female karyotypes (see **Supplementary Figure S1C**).

### Differentiation of hiPSCs into Mature Spinal Motoneurons

Motoneuronal differentiation of hiPSCs was performed as previously described by Hu and Zhang (2009). Modifications of the experimental protocol have been adopted from Stockmann et al. (2013). Motoneurons were fixed for immunochemical analysis after 21 and 42 days after final plating.

### Generation of Isogenic R495QfsX527^c.1483insC^

Clustered regularly interspaced short palindromic repeats (CRISPR) technology was used to generate an isogenic CNTL cell line derived from R495QfsX527 cell line, by inserting in position 1483 a cytosine (C), which is deleted in the patient cells, and consequently correcting the mutation.

Two sgRNAs (TATGATCGAGGCGGCTACCGGG, CGAGGGGGCCGGGGTGGTGGGG, Sigma-Aldrich) and a vector (Genewiz, UK) carrying the template (c-nucleotide, 731 bp up- and 715 bp downstream of the insertion site) were designed and transfected into a single iPS cells via human stem cell nucleofection kit using an AMAXA nucleofector (both from Lonza, Cologne, Germany). Cells were screened using a PCR based approach. After generation of isogenic CNTL line R495QfsX527^c.1483insC^, following CRISPR (**Supplementary Figure S2A**), the sequences upstream and downstream of the insertion site were sequenced to exclude additional alterations. Except for the cytosine insertion (highlighted in red) and the two proto-spacer adjacent motifs (PAM, highlighted in green), the alignment of the sequencing results of genomic CNTL and R495QfsX527^c.1483insC^ revealed no further alterations within the fragment (**Supplementary Figure S2B**).

### Immunocytochemistry

Immunochemical analysis were performed as previously described in standard protocols (Takahashi and Yamanaka, 2006; Aasen et al., 2008; Stockmann et al., 2013). HiPSCs and MNs were fixed by using 4% paraformaldehyde and 10% sucrose in phosphate buffered saline (PBS, Invitrogen). Primary antibodies were used under described incubation conditions: 1:1000 rabbit anti-FUS (Bethyl Labs, Montgomery, TX, USA); 1:2000 goat anti-TIA-1 (Santa Cruz Biotechnology, Heidelberg, Germany); 1:1000 chicken anti-tubulin beta-III (Millipore, Darmstadt, HE, Germany); 1:1000 chicken anti-NF-H (Antibodies online, Aachen, NRW, Germany); 1:1000 mouse anti-MAP-2 (Millipore, Darmstadt, HE, Germany); 1:1000 rabbit anti-γH2A.X (phosphor S139) (abcam, Cambridge, UK); 1:1000 chicken anti-ChAT (abcam), 1:1000 rabbit anti-Islet1 (ISL1) (abcam, Cambridge, UK); 1:300 mouse anti-(homeobox gen) (HB)-9 (DSHB, Iowa City, IA, USA), 1:200 rabbit anti-(nanog homeobox) (NANOG), 1:200 rabbit anti-OCT4, 1:200 rabbit anti-SOX2, 1:200 mouse anti-SSEA4, 1:200 mouse anti-(tumor-related antigen)-TRA1-60, 1:200 mouse anti-TRA1-81 (all Invitrogen) for 12 h at 4°C. Following fluorescence, labeled secondary antibodies were used: green Alexa Fluor^®^ 488, red Alexa Fluor^®^ 568, magenta Alexa Fluor^®^ 647 (all 1:500, Invitrogen). Glass cover slides were mounted with ProLong Gold Antifade reagent with DAPI (Invitrogen). Images were analyzed with a fluorescent microscope (Axiokop 2, Zeiss, Oberkochen, BW, Germany) using a CCD camera (16 bits; 1024 pixels per image) and the axiovision software (Zeiss).

### Western Blot

Western Blots were performed as previously described (Grabrucker et al., 2011). Protein concentration was determined by Bradford Assay; equal amounts of protein were separated using SDS-PAGE (polyacrylamide gel electrophoresis) and subsequently blotted on nitrocellulose membrane (GE Healthcare, Germany). Immunodetection of the primary antibody (Caspase-3, 1:500, Cell signaling, Danvers, MA, USA) was visualized by HRP-conjugated secondary antibody and ECL detection kit (Thermo Scientific). For quantification, Gel-analyzer Software 2010a was used. Subsequently, values were normalized against the loading control ß-actin (1:250.000, Sigma Aldrich. St. Louis, MO, USA).

### Induction of Cell Stress by Sorbitol and γ-Irradiation

Control and mFUS iPSCs were seeded on hESC-qualified-matrigel coated 13 mm glass cover slides (Menzel, Braunschweig, Germany) 1 day before treatment. To ensure a final concentration of 0.3 M, Sorbitol (Sigma-Aldrich, Schnelldorf, BW, Germany) was dissolved directly into pre-warmed mTeSR1 medium (Stemcell Technologies, Vancouver, CA, USA). Media was sterile-filtered and added to the cells followed by immediate incubation at 37°C for 30 min. Afterwards treated cells were fixed for immunostaining. In order to remove dead cells, medium was changed 1 h before treatment.

To analyze enhanced sensitivity of patient derived iPSCs to DNA damage, DNA breaks were induced by γ-irradiation (0.5 Gy) in control and mFUS hiPSC cell lines. Cells were lifted via hESC-dispase digestion (Stemcell Technologies) and transferred in suspension to a falcon tube. HiPSCs were then exposed to radiation and carefully seeded on hESC-qualified-matrigel coated 13 mm glass cover slides for immunochemical analysis. Subsequently, hiPSCs were analyzed after 24 h cultivation in mTeSR1 medium by quantifying cell colonies presenting normal/healthy or fragmented/apoptotic nuclei. To remove dead cells medium was changed 1 h before the fixation. All existing colonies from three cover slides were analyzed per experiment and condition. Subsequently, to determine the amount of double strand breaks after irradiation the samples were immunostained for rabbit anti-γH2A.X (abcam, 1:2000). To analyze, quantification of DNA damage foci positive cells were counted. Each experiment was repeated three times.

To analyze sensitivity of motoneurons expressing WT FUS or mFUS the neuronal spheres were plated on 35 mm dishes and kept in culture for 21/42 days. Medium was changed two times per week. The DNA breaks were induced by γ-irradiation (0.5 Gy) in control and mFUS MNs. Cells were fixed and analyzed after 24 h of cultivation by the quantification of DNA damage foci positive cells.

### Comet Assay

The comet assay was performed according to a standard protocol (Speit and Hartmann, 2006). HiSPC were lifted via hESC-dispase digestion (Stemcell Technologies) and transferred in suspension to a falcon tube. Afterwards iPSCs were exposed to γ-irradiation (0.5 Gy) and 10 μl cell suspension (about 10 000 cells) was mixed with 120 μl low melting point agarose (0.5% in PBS) and added to microscope slides (with frosted ends), which had been covered with a bottom layer of 1.5% agarose. Slides were then lysed (pH 10; 4°C) for at least 1 h. Slides were processed using alkali denaturation (at a pH > 13) for 25 min and followed by electrophoresis (0.86 V/cm). Images of 100 randomly selected cells stained with ethidium bromide were analyzed from each slide. Measurements were made by image analysis (Comet Assay IV, Perceptive Instruments, Haverhill, UK) and DNA migration was determined by measuring the “tail intensity” (% tail DNA). All samples were processed “double blinded” and analyzed by one researcher to reduce variability. Each experiment was performed independently three times, and the mean was calculated.

### Data Analysis and Statistics

For counting hiPS cells on each coverslip (*n* = 3) 3–10 representative images were taken randomly. Cells containing FUS positive inclusions under each condition (±sorbitol or ±irradiation) and the total number of cells labeled with DAPI were counted by using Image J Software^1^. Then, the mean of the positive cells/total number of cells ratio was calculated for each experiment (*n* = 3) and presented relative to untreated CNTL. Each specific type of colony (healthy, apoptotic, differentiated, and clumpy) ±irradiation was counted and expressed as a percentage of the total number of colonies relative to untreated CNTL. Cells positive for γH2A.X foci (either iPSC or MN) were counted and the number expressed as the percentage of the total number cells, in the same way as described for the number of cells containing FUS positive inclusions. According to the parameters, values were statistically analyzed either by one way ANOVA with a Bonferroni *post hoc* test to compare different cell lines or a unpaired *t*-test to compare two different conditions as indicated in each figure.

For quantification of FUS and TIA1 foci along neurites the “Find Foci plugin” (ImageJ) was used, which has previously been shown to closely match human assignments and reduce human inconsistencies in foci detection (Herbert et al., 2014). FUS, which co-localized with TIA1 foci were selected using a custom written program. Those foci could then be re-analyzed separately using “Find Foci.” In order to increase performance, spatial co-localization analysis was implemented using Python 3.5.1 in the Anaconda distribution. Imaging features of the scikit-image module (Van Der Walt et al., 2014) were used for loading and saving of image files. The masks created by the find foci plugin were processed. Masks of the FUS and TIA channel were processed pairwise. Every selection area in the first mask (FUS) was checked for a corresponding area in the second channel (TIA1). If such an overlap was found, both signals were deemed as spatially co-localizing. Non-co-localizing selections were removed and two new output masks were saved: a TIA1 mask and a FUS mask. These new masks only selected signals when at least a partial overlap with the other channel was found. They were used as input masks for “find foci” in order to analyze only these signals. The program was processed into a distributable file using PyInstaller. Single neurites (15–21) from five different cultures were analyzed per cell line. For Intensity measurements a minimum of 66 cells from three different cultures was analyzed. The signal intensity was quantified using ImageJ. An unpaired *t*-test was performed to compare CNTL versus mFUS.

All fluorescence images were obtained with an upright Axioscope microscope equipped with a Zeiss CCD camera (16 bits; 1280 ppi × 1024 ppi) using Axiovision software (Zeiss). All statistical analyses were performed using GraphPad Prism 5 (GraphPad Software Inc., La Jolla, CA, USA) software. Results are represented as mean values ± SEM. Statistical significance levels were set to *p* = 0.05.

### Ethics Statement

All procedures with human material were in accordance with the ethical committee of the Ulm University (Nr. 0148/2009 or 265/12) and Technische Universität Dresden (EK45022009) and in compliance with the guidelines of the Federal Government of Germany (Nr. O.103). The use of human material was approved by the Declaration of Helsinki concerning Ethical Principles for Medical Research Involving Human Subjects (Stockmann et al., 2013). All participants gave informed consent for the study.

## Results

### Characterization of hiPSC Lines

We generated and differentiated patient-derived cells lines (FUS1, FUS2, and FUS3) carrying three different endogenous mutations (all heterozygous) within the *FUS* gene. FUS1 harbored a “mild” missense mutation (R521C) with a late onset (57 years old), family history of ALS (Japtok et al., 2015), and a disease duration of 7 months. Cell line FUS2 carried the “malign” novel 1 bp deletion c.1483delC leading to a frameshift and the translation of 33 “new” amino acids before the STOP codon (R495QfsX527; Belzil et al., 2012; Japtok et al., 2015; Lenzi et al., 2015). This mutation presented with a juvenile ALS onset (27 years old) and death 16 months after onset. The patient-derived cell line FUS3 carried the most severe frameshift mutation c.1504delG. This leads to a modification of the FUS protein-coding sequence of exon 14 and 15 within a highly conserved region (RGG-rich). In addition and due to the loss of a functional stop codon, the translation of the protein is extended into the 3′-UTR (Asp502Thrfs^∗^27). This mutation leads to an early juvenile onset of ALS (19 at disease onset). All patients had a spinal onset, to a bulbar disease progression for FUS1 and FUS2. Patient-derived cell lines were compared with three control cell lines (CNTL1, CNTL2, and CNTL3) derived from neurologically healthy volunteers (male, age 29; female, age 45; male, age 79). Moreover, we corrected the mutation of the FUS2 line by CRISPR technology (**Supplementary Figure S2**) to test for mutation specific phenotypes (isogenic CNTL cell line R495QfsX527^c.1483insC^) (**Table 1**). All established hiPS cell lines (Asp502Thrfs^∗^27 is shown as an example) were checked for the expression of characteristic pluripotency markers including the nuclear factors OCT4, SOX2, NANOG and SSEA-4,TRA-1-60 and TRA-1-81 (**Supplementary Figures S1A,B** or as previously published (Japtok et al., 2015; Naujock et al., 2016). To confirm the ability of iPSCs to differentiate into ectoderm, endoderm, and mesoderm, specific markers were analyzed by immunocytochemistry including tubulin-ß-III, actinin, and AFP (**Supplementary Figure S1C**). Similarly, the generated iPSC line R495QfsX527^c.1483insC^ was positive for specific pluripotency markers. R495QfsX527^c.1483insC^ expressed the nuclear factors (all green) SOX2, OCT4 and NANOG and the surface markers (all red) SSEA-4, TRA1-60 and TRA1-81. The generated cell line showed high endogenous mRNA levels for *SOX2*, *OCT4*, and *NANOG*, whereas *KLF4* showed lower expression levels (**Supplementary Figure S2C**). In addition, cell lines presented normal karyotypes as shown in **Supplementary Figure S1D**.

**Table 1:** Human control and mFUS iPSC lines harboring different mutations of the *FUS* gene.

Cell line	Gender	Age^∗^	Mutation	Clinical	Additional information
FUS1	Female	57 (58) (58)	R521C, missense	Spinal Onset (arms), rapid progression to bulbar, disease duration 7 months died from respiratory failure	Family history of ALS with different phenotypes
FUS2	Male	26 (28) (28)	R495QfsX527 (c.1483delC), frameshift	Spinal onset (legs), upper and lower motoneuron involvement, hyperintense pyramidal tract in brain MRI; loss of walking ability 5 months after symptom ; non-invasive ventilation 10 months after symptom onset; death 16 months after onset	Juvenile ALS; *de novo* FUS mutation, both parents were tested negative
FUS3	Male	19 (20) (n.a)	Asp502ThrfS^∗^27 (c.1504delG), frameshift	Spinal onset (right hand, spreading to right leg, bulbar involvement during disease course); predominantly lower motoneuron involvement	Juvenile ALS, *de novo* FUS mutation, both parents were tested negative
CNTL1	Male	29	–		–
CNTL2	Female	45	–		–
CNTL3	Male	79	–		Father of FUS2
ISOGENIC CNTL	Male	26	R495QfsX527 c.1483InsC		Corrected mutation FUS2

*^∗^Age at onset (age at keratinocytes preparation/biopsy) (age of death)*.

### HiPSCs Expressing mFUS Are More Sensitive to Hyperosmolar Stress and Induced DNA Damage

In hiPSCs of control cell lines expressing WT FUS and hiPSC expressing mFUS, FUS was localized predominantly in the nucleus (**Supplementary Figure S1E**). Low levels of cytoplasmic FUS were detectable in some cells of the control cell lines CNTL1 and CNTL2 as well as in the cell lines carrying the R521C and R495QfsX527 mutations, but to a negligible degree (see arrowheads). Isogenic CNTL cell line R495QfsX527^c.1483insC^ confirmed this observation and showed also predominantly nuclear FUS (**Supplementary Figure S2D**). However, even at this early iPSC stage, some cells within the iPSC colonies from the mFUS cell line Asp502Thrfs^∗^27 exhibited a stronger cytosolic FUS mislocalization when compared to the other cell lines (**Supplementary Figure S1E**).

**FIGURE 1:**
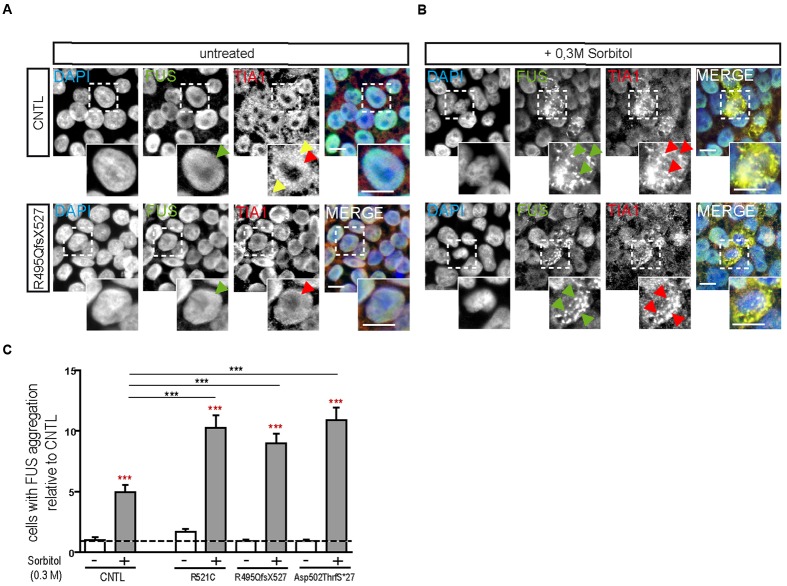
**Hyperosmolar stress induces cytoplasmic FUS positive SGs in mFUS-hiPSCs**. FUS immunostainings showing the nuclear distribution of wildtype and mFUS protein (green) in hiPSC lines **(A)** before and **(B)** after hyperosmolar stress conditions. In control and mFUS-hiPSC lines, FUS^+^ granules as well as TIA1^+^ SGs were detectable after stress. TIA1^+^ SGs are immunopositive for FUS as indicated by arrowheads. **(C)** Quantification of the amount of cells containing FUS^+^/TIA1^+^ SGs in mFUS cell lines relative to control. Statistical analysis revealed that after hyperosmolar stress all cell lines show a significant increase in FUS^+^ aggregates indicated by red asterisks (CNTL- vs. CNTL+ *p* ≤ 0.001, R521C- vs. R521C+ *p* ≤ 0.001, R495QfsX527- vs. R495QfsX527+ *p* ≤ 0.001, Asp502ThrfS^∗^27- vs. Asp502ThrfS^∗^27+ *p* ≤ 0.001). Unpaired *t*-test to compare untreated vs. treated; indicated by red asterisk. The increase was significantly higher in the mFUS cell lines (CNTL+ vs. R521C+, R495QfsX527+, Asp502ThrfS^∗^27+ *p* ≤ 0.001). Statistically significant differences were determined by one way ANOVA with Bonferroni *post hoc* test to compare individual groups. ^∗^*p* ≤ 0.05, ^∗∗^*p* ≤ 0.01, ^∗∗∗^*p* ≤ 0.001, + = sorbitol (0.3 M), CNTL = control, mFUS = mutated FUS, SGs = stress granuls. Scale bars: 10 μm.

Hyperosmolar stress was induced by exposing the hiPSCs to 0.3 M sorbitol for 30 min. Unstressed hiPSCs showed typical stem cell morphology with oval nuclei lying close to each other. Cells expressing WT or mFUS displayed a predominantly nuclear FUS localization in the absence of sorbitol or following mock-treatment (**Figure 1A**, green arrowheads). In addition, in unstressed control cells the SG marker TIA1 was found within nuclei (red arrowheads) and in some cells in the cytoplasm (yellow arrowheads, **Figure 1A**). In patient-derived cell lines expressing mFUS R521C, R495QfsX527 and Asp502ThrfS^∗^27 the TIA1 signal was also mainly nuclear without any cytoplasmic localization (**Figure 1A**). After hyperosmolar stress, however, FUS was seen in the cytoplasm forming several granules (green arrowheads, **Figure 1B**). This was seen in the control cell line as well as in all mFUS expressing cell lines. FUS^+^ granules perfectly co-localized with TIA1 (red arrowheads) indicating a specific localization of FUS to SGs (**Figure 1B**). Quantification of cells containing FUS^+^ cytoplasmic inclusions showed that in unstressed hiPSCs lines a very low proportion of cells displayed FUS aggregation and there were no significant differences between control and patient-derived cell lines (**Figure 1C**). After treatment with sorbitol, all cell lines responded to the treatment by having a significant proportion of cells containing FUS^+^ granules. This increase was 5.65 ± 0.42 fold in the control cell line CNTL and 6.02 ± 0.61, 9.50 ± 0.81, and 11.8 ± 1.12 fold in the mFUS cell lines R521C, R495QfsX527 and Asp502Thrfs^∗^27, respectively (*p* ≤ 0.001, unpaired *t*-test to compare non-treated versus treated cells within each cell line; red asterisk) (**Figure 1C**). The comparison of FUS/TIA1 inclusions of mFUS lines with controls after sorbitol treatment revealed a highly significant increase (CNTL vs. R521C, R495QfsX527 and Asp502Thrfs^∗^27 *p* ≤ 0.001, one way ANOVA with Bonferroni *post hoc* test to compare different groups).

**FIGURE 2:**
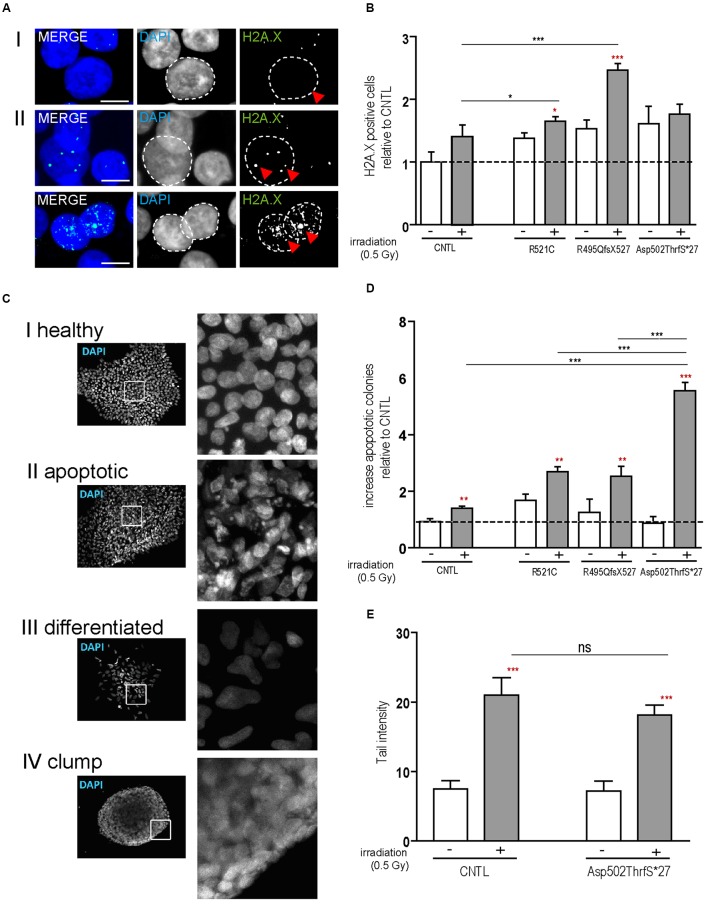
**mFUS-hiPSC are highly sensitive to induced DNA damage by irradiation. (A)** Classification of γH2A.X foci in hiPSCs from both controls or mFUS: **(I)** no foci **(II)** low and high level of foci. A representative image of Asp502ThrfS^∗^27 is shown. **(B)** The number of cells showing γH2A.X foci were nearly identical in all groups before irradiation; after irradiation the cell lines R495QfsX527 and R521C showed a significant increase of γH2A.X^+^ cells. Moreover, the R495QfsX527 line showed more affected cells than irradiated control and irradiated R521C (unpaired *t*-test to compare non-irradiated versus irradiated conditions within each cell line was performed; indicated by red asterisks, R521- vs. R521C+ *p* ≤ 0.05 and R495QfsX527- vs. R495QfsX527+ *p* ≤ 0.001). The effect of irradiation between the cell lines was analyzed by one way ANOVA with Bonferroni *post hoc* test to compare different groups, CNTL+ vs. R495QfsX527+ *p* ≤ 0.01 and R521C+ vs. R495QfsX527+ *p* ≤ 0.05. **(C)** Classification of hiPSC colonies in: **(I)** healthy, **(II)** apoptotic, **(III)** differentiated, and **(IV)** clumpy. **(D)** Apoptotic colonies were increased after irradiation (unpaired *t*-test to compare non-irradiated versus irradiated cells within each cell line was performed; indicated by red asterisks, CNTL vs. CNTL+ *p* ≤ 0.01, R521C vs. R521C+ *p* ≤ 0.01, R495QfsX527 vs. R495QfsX527+ *p* ≤ 0.01, Asp502ThrfS^∗^27 vs. Asp502ThrfS^∗^27+ *p* ≤ 0.001) and apoptosis was highly elevated in mFUS cells compared to control. The amount of differentiated and clumping colonies showed no differences after irradiation (data not shown). Analysis of significance was performed by one way ANOVA with Bonferroni *post hoc* test to compare different groups (CNTL+, R521C+ and R495QfsX527+ vs. p.Asp502Thrfs^∗^27+ ^∗∗∗^*p* ≤ 0.001). **(E)** The tail intensity (Comet assay after irradiation) of cell line CNTL and p.Asp502Thrfs^∗^27 was significantly increased as indicated by red asterisks but showed no differences between the control and mFUS cell line (one way ANOVA with Bonferroni *post hoc* test CNTL- vs. CNTL+ and Asp502ThrfS^∗^27- vs. Asp502ThrfS^∗^27+ *p* ≤ 0.001). ^∗^*p* ≤ 0.05, ^∗∗^*p* ≤ 0.01, ^∗∗∗^*p* ≤ 0.001, + = irradiation (0.5 Gy), CNTL = control, mFUS = mutated FUS. Scale bars: 10 μm.

Next we analyzed the susceptibility of hiPSCs specifically to DNA damage and repair by the induction of DNA breaks (γ-irradiation). DNA damage in hiPSC colonies was measured by immunolabeling γH2A.X positive foci (phosphorylated protein is recruited to DNA double strand breaks as depicted in **Figures 2AI,II**), which are proportional to the amount of DNA breaks (Fillingham et al., 2006). Cells were analyzed by counting the percentage of nuclei containing γH2A.X foci before and 24 h after irradiation. At base line, all cell lines presented a low number of the cells with γ-H2A.X foci but 24 h after DNA damage induction a significant increase was detected in the mFUS lines R521C (1.6 ± 0.07, *p* ≤ 0.05) and R495QfsX527 (2.464 ± 0.107 *p* ≤ 0.01). No increase was seen in the control cell line (1.4 ± 0.1) (**Figure 2B**) indicating that in the mFUS cell lines a higher number of DNA breaks was still detectable after 24 h of repair time (CNTL vs. R495QfsX527, *p* ≤ 0.01 and vs. R521C, *p* ≤ 0.05, one way ANOVA with Bonferroni *post hoc* test to compare different groups). The mFUS Asp502Thrfs^∗^27 line presented no significant increase, possibly due to a high amount of apoptotic cells as shown below. The specificity of this effect could be confirmed by the isogenic control line R495QfsX527^c.1483insC^. Quantification of γH2A.X positive cells before and 24 h after irradiation revealed that R495QfsX527^c.1483insC^ (1.36 ± 0.08, *p* ≤ 0.05) as well as R495QfsX527 (2.6 ± 0.11, *p* ≤ 0.05) showed an increased number of γH2A.X^+^ cells 24 h after DNA damage. This increase was much stronger in R495QfsX527 (*p* ≤ 0.001) expressing mFUS. Even without the induction of DNA damage, an increased amount of γH2A.X positive cells could be detectable for R495QfsX527 (1.65 ± 0.15, *p* ≤ 0.01) compared to R495QfsX527^c.1483insC^ (**Supplementary Figure S2F**). Next, we classified the hiPSC colonies according to morphological alterations after irradiation as being: (I) “healthy” (oval nuclei, lying tightly next to each other), (II) apoptotic colonies, (III) colonies with differentiated cells, and (IV) colonies forming clumps due to poor adherence (**Figure 2C**). Twenty-four hours after irradiation, all mFUS cell lines responded with a significant increase in apoptotic colonies (**Figure 2D**). The significant increase in the number of apoptotic colonies after irradiation was 1.3 ± 0.2 fold (*p* ≤ 0.01) in the control cell line and 1.0 ± 0.1 (*p* ≤ 0.01) and 2.0 ± 0.2 (*p* ≤ 0.01) fold in R521C and R495QfsX527, respectively. A highly significant increase (6.0 ± 0.3 fold, *p* ≤ 0.001) of apoptotic colonies was seen in the Asp502Thrfs^∗^27 line, in which most of the colonies were apoptotic after irradiation (**Figure 2D**). Unpaired *t*-test was used to compare non-irradiated versus irradiated cells within each group (red asterisks) and a one way ANOVA with Bonferroni *post hoc* test to compare the different groups. The number of differentiated and clumping colonies after irradiation was similar in the cell lines (data not shown). Finally, we evaluated the amount of DNA breaks by the comet assay. To this end, single cells of the colonies were γ-irradiated and the assay was performed directly thereafter. The CNTL as well as the mFUS cell line showed an increase in tail intensity after irradiation, however, no significant differences were seen between these lines (**Figure 2E**).

**FIGURE 3:**
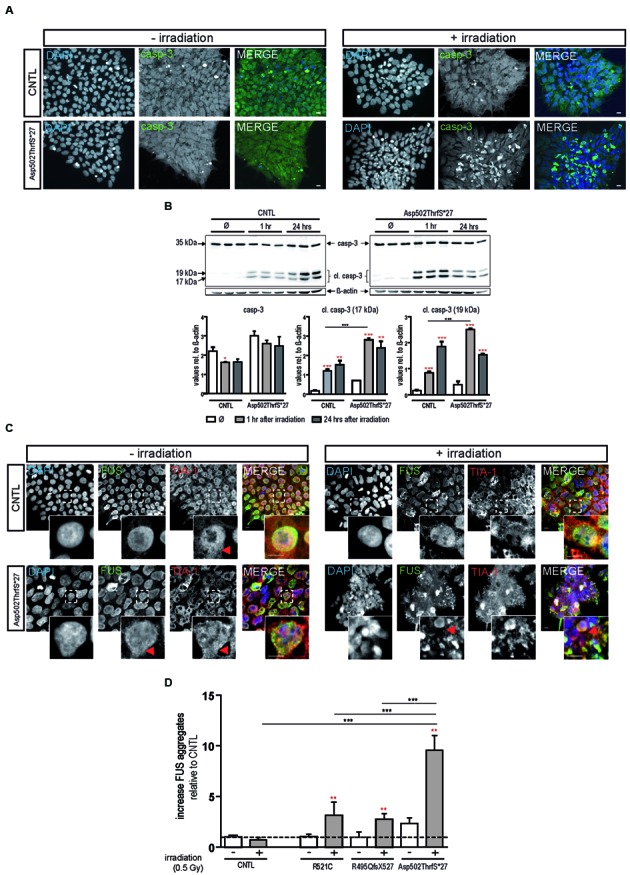
**Irradiation induces cytoplasmic FUS positive SGs and early apoptosis in mFUS-hiPSCs. (A)** Undifferentiated control and mFUS-hiPSCs were immunostained for caspase-3 (casp-3), after irradiation. In control and mFUS-hiPSCs caspase-3 positive cells were found within the hiPSC colonies. After irradiation the number of caspase-3 positive cells within colonies was increased, especially in the mFUS line Asp502ThrfS^∗^27 cell line. **(B)** Western Blot analysis of control and Asp502ThrfS^∗^27 hiPSCs 1 and 24 h after irradiation revealed a significant increase caspase-3 cleavage to cl.casp-3 (17 kDa) and cl.casp-3 (19 kDa) 1 h after the induction of DNA damage in mFUS Asp502ThrfS^∗^27 compared to control. The amount of full-length caspase-3 decreased after irradiation for control and mFUS. A significant reduction was seen in control 1 h after irradiation (casp-3 CNTL- vs. CNTL 1 h *p* ≤ 0.05). The amount of cleaved caspase-3 (17 kDa and 19 kDa) increased 1 and 24 h after irradiation for control and mFUS (unpaired *t*-test to compare untreated versus irradiated cells was performed; indicated by red asterisks). In comparison to control the values for the cleaved caspase 3 products were clearly enhanced in Asp502ThrfS^∗^27 cells. This effect was strongly significant after 1 h of irradiation. (one way ANOVA with Bonferroni *post hoc* test). Values are shown relative to ß-actin. **(C)** HiPSCs were immunostained for FUS (in green) and the SGs marker TIA1 (red) before and after irradiation. In control and mFUS-hiPSC lines FUS positive granules as well as TIA1 positive SGs were especially detected in the mFUS Asp502ThrfS^∗^27 cell line. Arrowheads indicate areas of FUS^+^/TIA1^+^ SGs co-localization in the cytoplasm. **(D)** Quantification of these FUS aggregates after irradiation shows a significant increase in all mFUS cells lines but not in CNTL. (unpaired *t*-test to compare non-irradiated versus irradiated cells from each cell line was performed; indicated by red asterisks; groups were compared by one way ANOVA with Bonferroni *post hoc* test. ^∗^*p* ≤ 0.05, ^∗∗^*p* < 0.01, ^∗∗∗^*p* ≤ 0.001, + = irradiation (0.5 Gy), CNTL = control, mFUS = mutated FUS, SGs = stress granules. Scale bar: 10 μm.

To confirm apoptosis induced by irradiation we also immunostained for caspase-3 and found that caspase-3 positive cells remained low in colonies from control cell line before and after irradiation but increased substantially in cells derived from the Asp502Thrfs^∗^27 cell line (**Figure 3A**). To quantify the amount of caspase-3 activation, caspase-3 and the two cleaved fragments (cl.casp-3 17 kDa and cl.casp-3 19 kDa) were analyzed via western blot 1 and 24 h after irradiation. The amount of full-length caspase-3 slightly decreased 1 and 24 h after irradiation for CNTL (0.73 ± 0.01 fold after 1 h, 0.74 ± 0.07 fold after 24 h) and mFUS (0.86 ± 0.05 fold after 1 h, 0.82 ± 0.15 fold after 24 h). Unpaired *t*-test was performed to compare untreated versus irradiated cells (red asterisks). Analysis of cleaved caspase-3 revealed a significant increase in cl.casp-3 (17 kDa) and cl.casp-3 (19 kDa) 1 h after the induction of DNA damage in mFUS Asp502ThrfS^∗^27 compared to the CNTL cell line. This increase after 1 h was 7.5 ± 0.52 fold in the CNTL cell line for cl.casp-3 (17 kDa) and 5.08 ± 0.3 fold for cl.casp-3 (19 kDa). Cell line Asp502Thrfs^∗^27 showed a 4.2 ± 0.1 fold increase in cl.casp-3 (17 kDa) and 6.59 ± 0.06 fold in cl.casp-3 (19 kDa) (**Figure 3B**).

In parallel, hiPSCs were also co-immunostained for FUS and TIA1 before and 24 h after irradiation. In non-irradiated cells, FUS was mainly distributed in the nuclei, however, the cell line Asp502Thrfs^∗^27 presented a certain degree of cytoplasmic FUS (**Figure 3C**, red arrowheads). TIA1 staining was also mainly nuclear with some cytoplasmic staining (**Figure 3C** insert, red arrowheads). After DNA damage we found a significant increase in the number of cells that contained cytoplasmic FUS forming aggregates that were positive for TIA1 (**Figure 3C**). In all mFUS cell lines, the number of cells with FUS/TIA1 cytoplasmic inclusions was significantly increased after irradiation. The most pronounced effect was seen in the cell line with the Asp502Thrfs^∗^27 mutation with an significant increase of 9.5 ± 0.1.4 fold (*p* ≤ 0.001) as opposed to increases of 3.3 ± 1.3 (*p* ≤ 0.01) and 2.7 ± 0.5 (*p* ≤ 0.01) fold in R521C and R495QfsX527, respectively and no significant increase in the control cell line (0.7 ± 0.2 fold, *p* > 0.05) (**Figure 3D**). Unpaired *t*-test was used to compare non-irradiated versus irradiated cells within each group (red asterisk), one way ANOVA with Bonferroni *post hoc* test to compare different lines).

### Cytoplasmic FUS Mislocalization Appears during Neuronal Aging and Depends on the Severity of the FUS Mutation in Spinal Motoneurons

Motoneurons differentiated from hiPSCs were tested for spinal MN differentiation by immunostaining with antibodies directed against TUJ1 (beta-III tubulin), NF-H, HB9, Islet-1 (ISL-1), and ChAT (**Supplementary Figure S3A**). On day 21 of motoneuron differentiation all cell lines developed a dense neuronal network and expressed the early neuronal protein marker beta-III tubulin (**Supplementary Figure S3B**). At this stage, HB9 and Islet-1 (transcription factors specific for MNs) as well as ChAT (characteristic for cholinergic MN) were also detectable (**Supplementary Figure S3A**). At day 42, the neuronal network had matured in all cell lines and contained dense NF-H positive filaments (**Supplementary Figure S3C**).

**FIGURE 4:**
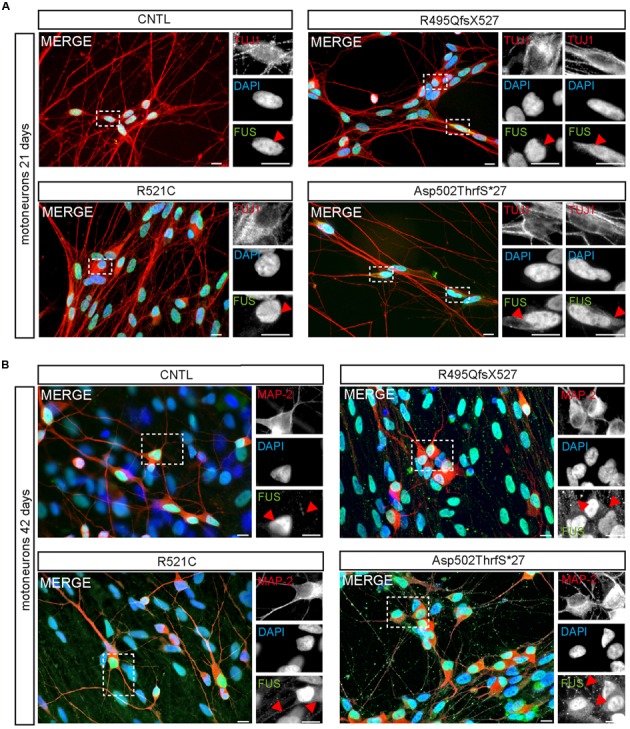
**Cytoplasmic FUS mislocalization depends on motoneuron aging and FUS mutation type**. **(A,B)** Immunostaining of FUS (green) in spinal motoneurons at different stages of motoneuronal development. **(A)** In TUJ1 positive (red) 21 days old motoneurons FUS is predominantly found in the nuclei. Little spots of cytoplasmic FUS was detectable only in cells expressing Asp502ThrfS^∗^27 and R495QfsX527 FUS. **(B)** In MAP2 positive (red) 42 days old control motoneurons FUS is still confined to the nuclei. Cytoplasmic FUS and FUS positive granules along neurites were detected in R521C, R495QfsX527 and Asp502ThrfS^∗^27 derived motoneurons. Motoneurons with the malign R495QfsX527 and severe Asp502Thrfs^∗^27 FUS mutation presented larger FUS deposits within the cytoplasm compared to the R521C cell line. Scale bars: 10 μm.

Within developing and mature MN, FUS was predominantly localized in the nucleus of all 21-day-old MNs (**Figure 4**). Similar to controls, neurons carrying the benign missense mutation R521C exhibited an exclusive nuclear localization of the FUS protein. However, cytoplasmic FUS was detectable in some MNs expressing mFUS with the frame shift mutation R495QfsX527. In contrast, almost every cell harboring the most severe mutation Asp502Thrfs^∗^27 contained cytoplasmic FUS (**Figure 4A**). To visualize motoneuronal cell bodies as well as dendrites, differentiated cells were stained for MAP-2 (**Figure 4B**).

### FUS Granules Are Regularly Found in Neurites of iPSC Derived MN

**FIGURE 5:**
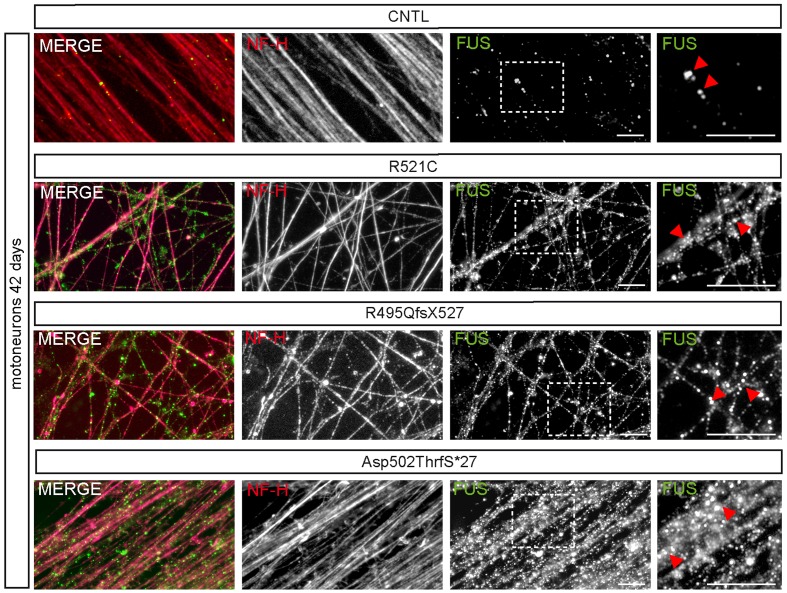
**mFUS-hiPSCs derived motoneurons are characterized by an increased number of large FUS^+^ granules along neurites**. Immunostaining of FUS (green) in mature 42-day-old motoneurons showed FUS positive granules along NF-H^+^ (magenta) neurites in control and mFUS-hiPSCs derived cells. FUS positive granules or small punctae were observed regularly in control cells; in mFUS motoneurons several, larger FUS positive granules were detected along the neurites. mFUS = mutated FUS, scale bars: 10 μm.

At day 42 of neuronal differentiation, FUS was localized to the nucleus of control cells (**Figure 4B**), in patient-derived cells, the protein was in addition detectable in the cytoplasm of almost every neuronal cell carrying mFUS R521C, R495QfsX527 and Asp502Thrfs^∗^27. In particular, MNs with the mFUS R521C displayed some FUS protein in the cytoplasm as well as along dendrites (**Figures 4B** and **5**). MNs carrying the malignant FUS mutations R495QfsX527 or Asp502Thrfs^∗^27 showed a signal for FUS in cytoplasm and FUS inclusions within the cytoplasm and along the MAP-2 or NF-H positive neurites (**Figure 4B**, red arrowheads and **Figure 5**). FUS^+^ punctae were especially seen in neurites of patient-derived MN, especially Asp502Thrfs^∗^27 derived MNs had an aberrant and an increased amount of large FUS^+^ punctae (**Figure 5**, red arrowheads). In contrast to MNs expressing the mFUS variant R495QfsX527 the CRISPR corrected cell line R495QfsX527^c.1483insC^ showed only nuclear FUS in differentiated MN (**Supplementary Figure S2E**, red arrow heads).

**FIGURE 6:**
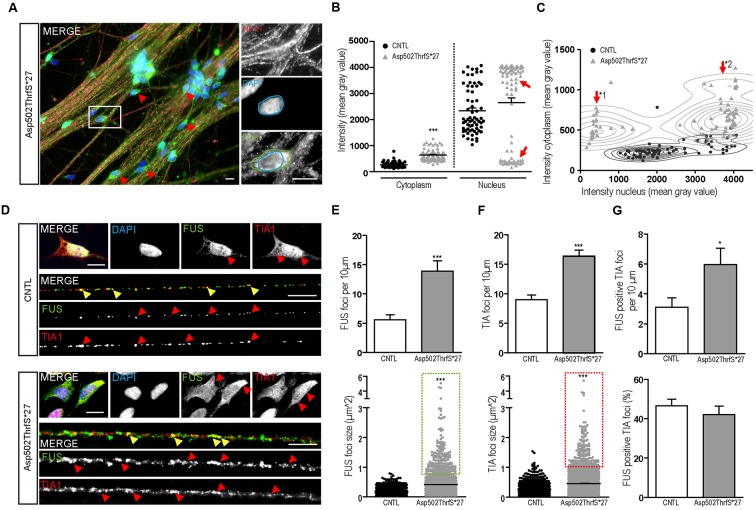
**Characterization of FUS expression and localization in mFUS motoneurons**. **(A)** Mature, 42-day-old motoneurons derived from mFUS Asp502ThrfS^∗^27 were immunostained for FUS (green) and NF-H (magenta) to identify FUS alterations associated with the severity of the FUS mutation. In some cells a drastic mislocalization could be identified (as indicated by red arrowheads). In those motoneurons, a large number of FUS^+^ granules along the neurites were detectable and FUS was almost completely absent from the nuclei (blue circle) and found strongly positive in the cytoplasm (green circle). **(B)** Quantification of this observation by intensity measurements (mean gray value) revealed significant values for cytoplasmic FUS in Asp502ThrfS^∗^27 motoneurons compared to control (unpaired *t*-test). In control, the measurements of nuclear areas resulted in one homogenous cell population; in Asp502ThrfS^∗^27 motoneurons two separated cell populations were identified (red arrows). **(C)** These two cell populations are further characterized in a diagram, showing individual values for cytoplasmic (*y*-axis) and nuclear (*x*-axis) intensity measurements of the analyzed motoneurons. The two Asp502ThrfS^∗^27 cell populations, which clearly differ in the nuclear amount of FUS, are highlighted by ^∗^1 and ^∗^2. **(D)** Mature spinal motoneurons were stained for FUS (green) and TIA1 (red). FUS was detectable in the nuclear compartment in control cells, cytoplasmic FUS was detectable only in motoneurons expressing mFUS. In control and mFUS motoneurons, TIA1 was seen in the nucleus but also in cytoplasm. Neurites from 42 days old spinal motoneurons showed FUS^+^ granules in control and in mFUS cells. The number of those granules were largely increased in motoneurons expressing mFUS. Moreover, TIA1^+^ SGs were detectable in control but to a higher degree and increased size in mFUS-hiPSCs derived neurites. FUS^+^ and TIA1^+^ foci are shown per 10 μm neurite length **(E)** Quantification of FUS^+^ foci showed a significant increase in the number of foci in mFUS cells. The size of the FUS^+^ foci was also increased in Asp502ThrfS^∗^27, as highlighted by the green insert. **(F)** Quantification of TIA^+^ SGs revealed an increase in the granules number along the neurites in mFUS, that were also larger compared to control as highlighted by the red insert. **(G)** Co-localization analysis showed that there is an increased degree of FUS^+^ TIA1 foci per 10 μm in Asp502ThrfS^∗^27. The overall percentage of co-localized FUS and TIA1, however, is similar in control and mFUS. ^∗^*p* ≤ 0.05 and ^∗∗∗^*p* ≤ 0.001; SGs = stress granules; CNTL = control; and mFUS = mutated FUS. Scale bars: 10 μm.

Interestingly, several mature 42-day-old MN derived from cell line Asp502Thrfs^∗^27 showed FUS immunoreactivity that was almost excluded from the nucleus and shifted completely to the cytoplasm (**Figure 6A**). To quantify the amount of cytoplasmic and nuclear FUS, we measured the intensity (mean gray value) of FUS in the cytoplasm and/or in the nuclei of NF-H positive MN (**Figures 6B,C**). In controls the homogenous cell population showed low values for the cytoplasm and high intensities for the nuclei. In MN carrying the Asp502ThrfS^∗^27 mutation two separated MN populations could be identified according to their nuclear staining. They either had a very reduced nuclear FUS value (mean gray value of 452.1 ± 51.23, *p* ≤ 0.001) or a significantly higher intensity (mean gray value of 3759 ± 46.78, *p* ≤ 0.001) (**Figure 6B**, highlighted by arrow heads). In the cytoplasm, there was a significant higher amount of cytoplasmic FUS in Asp502ThrfS^∗^27 (mean gray value of 6393 ± 23.26) compared to CNTL (mean gray value of 263.3 ± 12.73, *p* ≤ 0.001).

The two populations of Asp502ThrfS^∗^27 MN were further characterized by a correlation analysis taking cytoplasmic and nuclear intensity of each single cell into account. In contrast to CNTL the mFUS cells were splitted into two populations with high cytoplasmic intensity and either low nuclear intensity (**Figure 6C**, arrow ^∗^1) or high nuclear intensity (**Figure 6C**, arrow ^∗^2). The thorough analysis of the first subgroup (^∗^1) displaying a high degree of cytoplasmic FUS were found to be also displacing large FUS^+^ granules along the neurites. Neurites derived from healthy CNTL showed 5.5 ± 0.8 FUS^+^ granules per 10 μm whereas Asp502Thrfs^∗^27 presented significantly more FUS^+^ granules (13.9 ± 1.7, *p* ≤ 0.001) (**Figures 6D,E**). In addition, in CNTLs the average size of the FUS^+^ granules was 0.17 ± 0.004 μm^2^, whereas the granules found in the mFUS Asp502Thrfs^∗^27 line where significantly larger (0.4 ± 0.013 μm^2^) (*p* ≤ 0.001) (**Figure 6E**, dot blot). Unpaired *t*-test was used to compare CNTL versus mFUS cells.

### mFUS Motoneurons Show an Increased Number of SGs along Neurites

In order to further characterize these FUS^+^ granules along mature neurites, the intracellular distribution of TIA1 positive SGs in 42 days old MN was investigated. Similar to cells expressing WT FUS, MNs carrying FUS mutations exhibited a predominantly nuclear expression of TIA1. In addition, TIA1^+^ inclusions were found along the neurites and partially co-localizing with FUS in control cell lines as well as in mFUS MN (indicated by yellow arrowheads in **Figure 6D**). In cells expressing mFUS, however, there were notably more and larger TIA1/FUS positive granules than in control MNs. Neurites derived from CNTL cell lines showed an average of 9.0 ± 0.8 TIA1^+^ SGs per 10 μm length whereas Asp502Thrfs^∗^27 presented a significant increase by having an average of 16.3 ± 1.05 TIA1 granules per 10 μm (*p* ≤ 0.001) (**Figure 6F**). The ratio of co-localization between FUS and TIA1 granules showed no differences (*p* > 0.05) between CNTL (46.6 ± 3.2%) and mFUS Asp502ThrfS^∗^27 (42.1 ± 4.2%) (**Figure 6G**), but in line with the elevated number of FUS^+^ granules *per se* there is a significant increase of FUS positive TIA1 foci in cells expressing Asp502ThrfS^∗^27 (5.9 ± 1.0) compared to CNTL (3.1 ± 0.6, *p* ≤ 0.05, unpaired *t*-test to compare CNTL versus mFUS) (**Figure 6G**).

### Motoneurons Expressing mFUS Are Characterized by Increased DNA Damage Foci

**FIGURE 7:**
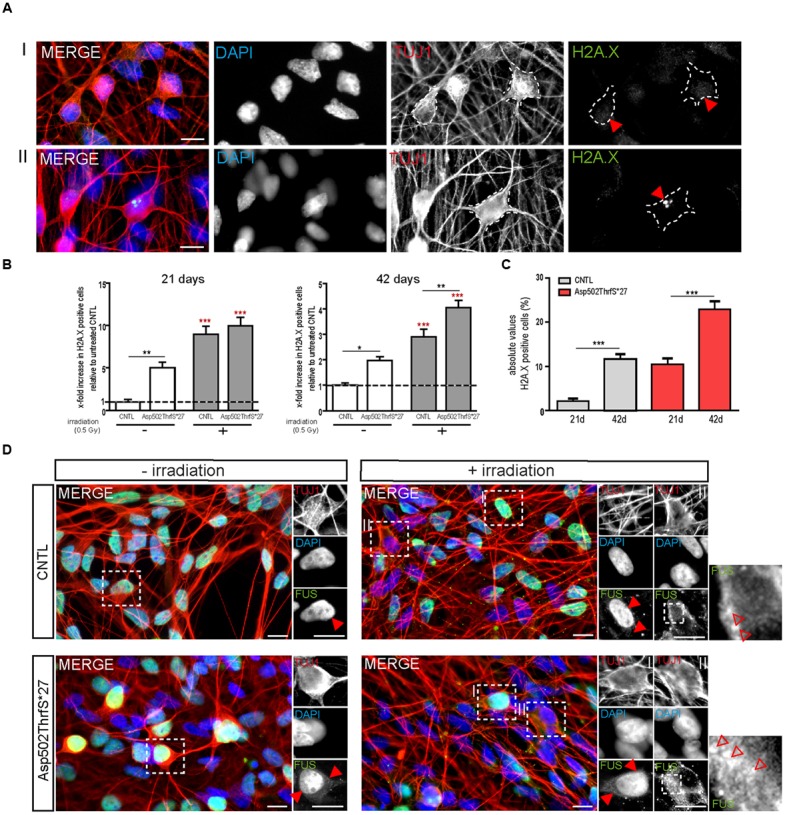
**mFUS motoneurons show signs of increased DNA damage which are potentiated after irradiation**. **(A)** 21 days old motoneurons before and 24 h after irradiation were immunostained by γH2A.X (green) and categorized either negative **(I)** or positive **(II)** for DNA damage. **(B)** Even before γ-irradiation the amount of DNA damage was significantly increased in 21 and 42 days old motoneurons expressing Asp502ThrfS^∗^27. After irradiation the control as well as the Asp502ThrfS^∗^27 cell line showed an increased number of γH2A.X^+^ cells compared to non-irradiated; 42 days old motoneurons expressing mFUS show more DNA damage compared to control. Results are displayed relative to the non-irradiated control line (unpaired *t*-test; comparison of cell lines was performed by one way ANOVA with Bonferroni *post hoc* test). **(C)** The effect of maturation (21 vs. 42 days old motoneurons) on γH2A.X^+^ cells revealed significant results for control as well as for Asp502ThrfS^∗^27 (unpaired *t*-test). **(D)** 21 days old cells before and 24 h after irradiation were stained for FUS. Without irradiation FUS is predominantly present in the nucleus in control and Asp502ThrfS^∗^27 cells. Some cytoplasmic FUS staining is seen in young mFUS cells. After irradiation, in control as well as Asp502ThrfS^∗^27 FUS is mislocalized to the cytoplasm (insert II, red arrowheads) in some motoneurons. In addition to this Asp502ThrfS^∗^27 motoneurons show large positive FUS inclusions (insert II, red arrowheads). ^∗^*p* ≤ 0.05, ^∗∗^*p* ≤ 0.01, and ^∗∗∗^*p* ≤ 0.001; CNTL, control and mFUS, mutated FUS. Scale bars: 10 μm.

As already performed for iPS cells, we next analyzed the amount of DNA-damage in young (21 days old) and mature (42 days old) MNs 24 h after γ-irradiation. MNs were classified either negative (I) or positive (II) for DNA damage foci as indicated by γH2A.X^+^ labeling (**Figure 7A**, red arrowheads). Interestingly, we found that even in non-stressed 21 days old MNs the number of cells containing γH2A.X^+^ foci was significantly higher in the mFUS Asp502Thrfs^∗^27 line (5.0 ± 0.6 fold) compared to control (1.0 ± 0.2, *p* ≤ 0.01). Both neuronal lines responded to irradiation by showing a significant increase in cells containing positive γH2A.X foci in the nuclei (8.9 ± 2.0 fold in CNTL and 9.9 ± 2.0 fold in Asp502Thrfs^∗^27, *p* ≤ 0.001; **Figure 7B**). This increase of γH2A.X positive neurons after irradiation was not significantly different between the two cell lines. Similar to young cells, the non-stressed 42 days old MNs expressing Asp502Thrfs^∗^27 exhibited a significant increased number of γH2A.X^+^ cells (1.9 ± 0.1 fold) compared to control (1.0 ± 0.09, *p* ≤ 0.05). After irradiation, all lines responded with an increase of γH2A.X positive neurons (2.9 ± 0.2 fold in CNTL and 4.0 ± 0.2 fold in Asp502Thrfs^∗^27, *p* ≤ 0.001). Unpaired *t*-test was used to compare non-irradiated versus irradiated within each cell line. However, different from the iPSCs (Asp502Thrfs^∗^27), the increase of DNA damage positive cells after irradiation was significantly increased for stressed mFUS compared to stressed CNTL (*p* ≤ 0.01, one way ANOVA with Bonferroni *post hoc* test to compare different lines before and after irradiation) (**Figure 7B**). A comparable result was seen using the isogenic control line R495QfsX527^c.1483insC^ underlining the assumption that mFUS is most likely responsible for the observed phenotype. Here, an effect of irradiation was only seen for R495QfsX527^c.1483insC^ showing an increase of 1.8 ± 0.12 (*p* ≤ 0.001, unpaired *t*-test) 24 h after irradiation. Again, differences between the mutated and rescued cell line was detectable even before and after irradiation of mature motoneurons. Compared to non-irradiated R495QfsX527^c.1483insC^, non-irradiated R495QfsX527 showed higher numbers of γH2A.X^+^ cells (2.8 ± 0.19, *p* ≤ 0.001). Although irradiation had no obvious effect on R495QfsX527 (2.7 ± 0.2), significantly more γH2A.X^+^ cells were detectable compared to irradiated R495QfsX527^c.1483insC^ (1.8 ± 0.12, *p* ≤ 0.001) (**Supplementary Figure S2F**).

Finally, we analyzed the number of DNA damage foci during motoneuronal maturation. Young 21 days old CNTL-MN showed only a 2.0 ± 0.6% of γH2A.X positive cells whereas 11.6 ± 1.0% γH2A.X positive cells were present in mature 42 days old cultures (*p* ≤ 0.001). DNA damage levels were generally increased in mFUS cells. Twenty-one days old Asp502Thrfs^∗^27-MN exhibited 10.4 ± 1.4% γH2A.X positive cells; whereas in 42 days old MN 22.9 ± 1.835% of the cells were positive for foci of DNA damage (*p* ≤ 0.001, unpaired *t*-test to compare 21 days versus 42 days) (**Figure 7C**). To study the effect of DNA-damage on FUS localization in young MN, we also stained for FUS. In young CNTRL-MN, FUS is localized in the nuclei, after irradiation FUS could partially be seen in the cytoplasm (**Figure 7D**). After irradiation of young mFUS-MN cultures large FUS clusters were detected in the cytoplasm in nearly all neurons compared non-stressed cells (**Figure 7D**, arrowheads).

## Discussion

Since the discovery of specific gene mutations leading to ALS, several model systems have been established to analyze pathomechanisms leading to a selective degeneration of MNs. With respect to FUS, there have been publications on different murine models with inconsistent pathologies. For example, transgenic mice overexpressing human WT FUS exhibited MN degeneration, an ALS hallmark, indicating a toxic effect of elevated FUS levels (Huang et al., 2011). However, in rats it was only the overexpression of mFUS and not WT FUS that induced motor deficits (Mitchell et al., 2013). Moreover, FUS inclusions, which are detectable in brains and spinal cords of ALS patients (Vance et al., 2009; Dormann et al., 2010) could be induced in rodents by overexpressing WT as well as mFUS (Huang et al., 2011). However, the phenotype was more severe and the inclusions more frequent in cells expressing mFUS, indicating that mFUS is more toxic to neurons. Therefore, it is tempting to speculate that mutations disrupting the protein structure may not only be completely responsible for the disease but also changes in FUS protein levels. In fact, mutations in the 3′-UTR of the *FUS* gene that lead to increased FUS levels through an altered feed-forward regulatory loop, are associated with ALS (Dini Modigliani et al., 2014), suggesting that FUS has to be properly regulated to avoid pathology. Non-neuronal cells, which do not recapitulate the complexity of neurons, as well as neurons have also been extensively used *in vitro* as valuable tools to analyze cell specific responses but only by overexpressing WT or mFUS (Dormann et al., 2010; Bentmann et al., 2012; Jäckel et al., 2014).

In our study, we took advantage of the hiPSC technology to generate patient specific cell lines, avoiding the overexpression of the protein, and test whether these cells were able to recapitulate ALS pathology in undifferentiated hiPSC and in selectively differentiated spinal MNs. We generated a novel iPS cell line from a juvenile ALS patient presenting with a frame shift FUS mutation in the RGG-rich region Asp502Thrfs^∗^27 (Hübers et al., 2015) and compared it with our previously described cell lines R521C and R495QfsX527 (Japtok et al., 2015). In iPS cells under physiological *in vitro* conditions FUS was only mislocalized in the cell line Asp502Thrfs^∗^27 but not in the other cell lines in which mFUS remained mainly nuclear.

The induction of hyperosmolar stress by sorbitol treatment, however, led to FUS^+^ granules seen in the cytoplasm co-localizing with the SGs marker and mRNA binding protein TIA1. The number cells containing cytoplasmic FUS^+^ granules after sorbitol treatment was significantly higher in cells expressing mFUS compared to controls. This increase was nearly identical in all mFUS cell lines, indicating that this phenotype was not associated with the severity of the underlying FUS mutation as it was shown for oxidative stress before (Lenzi et al., 2015). These observations recapitulate published data on the recruitment of FUS and mFUS into SGs as described in hiPSC and differentiated MN (Lenzi et al., 2015) and reviewed by (Sama et al., 2014a). This result might suggest a physiological or protective role of WT FUS within the stress response (Sama et al., 2014b), whereas mFUS^+^ SGs are more pathogenic. Since it is known that osmotic stress enhanced the degeneration of motoneurons in a *C. elegans* model of ALS (Therrien et al., 2013) this kind of stress condition may also contribute to ALS pathology by a transformation of reversible FUS positive SGs into pathological inclusions.

Fused in sarcoma has also been shown to be involved in DNA damage and DNA repair by interacting directly with HDAC1. As shown by Wang et al. in HEK cells, FUS is recruited to DNA repair sites allowing proper DDR signaling (Wang et al., 2013). Interestingly, these authors showed that impaired efficiencies in DNA repair might also be depending upon the site of mutation. In this study, we have tested the degree of DNA damage and repair in two types of human cells: (I) In hiPSC, which divide rapidly and show high levels of DNA repair and (II) in MN, which are postmitotic, slower DNA repairing cells. Interestingly, we found opposing results for these two cell types. In iPSCs, the degree of DNA damage was very low and no differences were found between control and mFUS cell lines. After the induction of double strand breaks by irradiation, DNA damage was increased but no differences between WT and mFUS were encountered as assessed by the comet assay directly after irradiation. However, differences between WT and mFUS cell lines are detectable at the morphological level when cells were left to repair for 24 h. The number of colonies who underwent apoptosis after irradiation was significantly higher in mFUS cells indicating that cells expressing WT FUS restore their DNA more efficiently compared to cells expressing a mFUS variant. This aspect could be substantiated by the analysis of active caspase-3 as an apoptotic marker protein. In the mutant R495QfsX527 we also detected an increase in the amount of cells containing DNA damage foci after 24 h of irradiation, indicating that this FUS mutation seems to cause an inefficient DNA repair. Reduced differences were seen for the mild mutation, demonstrating that this mutation may have a weaker effect (Wang et al., 2013). For the most aggressive mutation, the percentage of cells containing DNA damage foci was unchanged, but most likely because cells were already apoptotic and the DNA damage in apoptotic cells could not be assessed.

**FIGURE 8:**
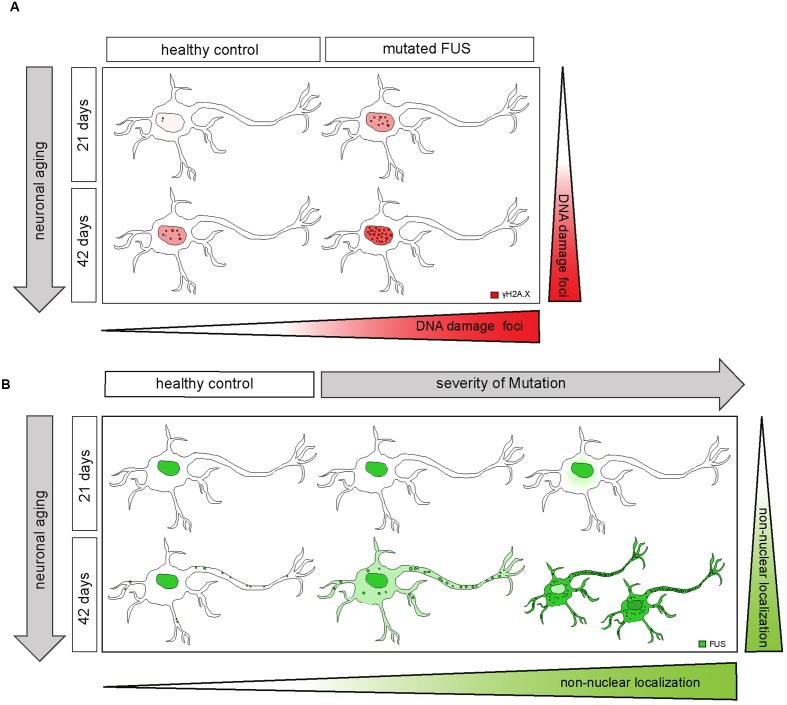
**Extra-nuclear FUS-localization depends on neuronal aging and severity of the mutation**. **(A)** Diagram showing how mFUS and neuronal aging influence the degree of DNA damage in differentiated motoneurons. There is an increase in DNA damage during aging in healthy control and mFUS cells. However, in young and mature motoneurons the accumulation of DNA damage is higher compared to control in each case. **(B)** Diagram showing how neuronal aging and the severity of mutation influence FUS localization in hiPSCs-derived motoneurons and thereby contribute to the formation of pathological FUS^+^ inclusions. Motoneurons derived from neuronal healthy volunteers expressing wildtype FUS present a predominantly nuclear FUS localization during early and later neuronal development. Furthermore, mature motoneurons show FUS along neurites within defined punctae. In motoneurons derived from ALS-FUS patients expressing mFUS the protein is also localized mainly nuclear during early neuronal development. In cells expressing mFUS with a severe mutation, cytoplasmic mislocalization starts earlier and is already detectable in 21 days old motoneurons. In mature mFUS cells (42 days), two separated cell populations, which differ in the nuclear amount of FUS are present, one with a higher amount of FUS and another one with FUS almost completed shifted to the cytoplasm. CNTL, control and mFUS, mutated FUS.

When we induced double strand breaks in spinal MN derived from FUS patients and healthy controls we tested the hypothesis that the induction of DNA damage could be a putative second hit leading to MN degeneration (Madabhushi et al., 2014). Interestingly, compared to iPSCs, which showed no differences in DNA damage in non-irradiated conditions, 21 days old MN expressing mFUS Asp502Thrfs^∗^27 exhibited a significantly increased number of DNA damage foci, suggesting the accumulation of DNA damage as an early event that might indeed induce disease pathology, furthermore, an accumulation of DNA damage foci was associated with aging of the MN (as represented in **Figure 8A**). Different studies have already shown the association of FUS function in DNA repair and genome instability. KO FUS mice die at birth and show genomic instability (Hicks et al., 2000) and fibroblasts derived from these mice are very sensitive to radiation (Hicks et al., 2000; Kuroda et al., 2000). FUS also induced the annealing of DNA-loops by homologous recombination, a crucial step in DNA repair (Naro et al., 2015). Similar, to what we detected in our mFUS derived MN, an accumulation of DNA damage was also seen in post mortem cortex sections of fALS patients harboring FUS mutations (Wang et al., 2013) and the spinal cord of mice expressing a FUS mutation (Qiu et al., 2014). Recently, it has also been shown that WT FUS is affected after DNA damage by being phosphorylated at the N terminus and being translocated into the cytoplasm (Deng et al., 2014). In our study, the irradiation of young MN from a control subject resulted in FUS protein translocated into the cytoplasm and therefore, mimicking pathological changes that occur in FTD-FUS and ALS-FUS. In mFUS-MN, irradiation induced an even more extreme phenotype by forming large FUS positive cytoplasmic inclusions. Altogether, the data suggest that the accumulation of DNA damage in mFUS may be an early, crucial event that triggers the pathological changes detected in neurodegeneration of MN in ALS related diseases (Madabhushi et al., 2014).

Finally, FUS is known to transport RNA from the nucleus to the cytoplasm being associated with several motor proteins (Kanai et al., 2004; Yoshimura et al., 2006; Takarada et al., 2009). In neurons, FUS is found at synaptic sites indicating that it might be important for local translation of transported mRNA (Fujii and Takumi, 2005; Fujii et al., 2005; Yasuda et al., 2013; Sephton et al., 2014) and involved in synaptic plasticity (Fujii et al., 2005; Aoki et al., 2012; Schoen et al., 2016). Therefore, we investigated the localization of FUS in control and patient-derived MNs and analyzed a FUS associated pathology in axons and/or dendrites. We have previously shown that iPSC-derived MNs express a wide set of synaptic markers after 42 days in culture and establish mature synaptic contacts with the ability to generate action potentials (Stockmann et al., 2013). In young, 21 days differentiated MNs, FUS was mainly localized in nuclei, except for the most aggressive mutation in which FUS was already translocated into the cytoplasm. In mature 42 days differentiated MN from control cell lines, FUS was not only localized in the nuclei (Japtok et al., 2015; Lenzi et al., 2015; Liu et al., 2015) but was also detectable as small punctae along the neurites. This observation was very similar to the FUS localization along neurites and within the presynaptic compartment in hippocampal neurons (Schoen et al., 2016).

In 42 days old MN from cell lines harboring mFUS, the protein was mainly seen in the nuclei but also distributed within the cell body at different degrees. This degree of mislocalization was directly associated with the clinical onset of the disease. The milder mutation R521C, with a late onset, presented only some FUS mislocalization, while the amount of cytoplasmic FUS distribution was higher in the other two most severe mutations. In these cases, small FUS^+^ inclusions were also detected within the cytoplasm (**Figure 8B**). In addition, in the cell line harboring the most severe mutation, with an early juvenile onset, we could identify two subgroups of MN with high amounts of cytoplasmic FUS and either low or high nuclear FUS levels. In these MN the degree of punctuated FUS along the neurites was much stronger and abundant compared to controls. Furthermore, some of these large FUS punctae co-localized with SG marker, which were also present along the neurites. This indicates, that in mFUS, FUS is spontaneously aberrantly distributed along the axons, as opposed to iPSCs, in which as stressor was needed to see changes in FUS mislocalization and SG formation (Lenzi et al., 2015; Ichiyanagi et al., 2016). It is quite conceivable that these mFUS clusters affect local mRNA translation at the synapse and contribute to the synaptic loss in ALS related neurodegenerative disease.

In summary, we have been able to reproduce in human iPSC derived MN some crucial pathological hallmarks seen in ALS such as (I) the cytoplasmic FUS mislocalization, (II) the formation of FUS^+^ inclusions (Japtok et al., 2015; Lenzi et al., 2015; Ichiyanagi et al., 2016) as well as (III) an increased DNA damage in MN (Wang et al., 2013; Qiu et al., 2014). Moreover, FUS was seen to build up large clusters in neurites, especially in mFUS cell lines carrying aggressive mutations. Undifferentiated hiPSCs were less sensitive to mFUS and did not show an obvious cellular phenotype. This could, however, be induced by specific stressors, and mFUS cell lines were more vulnerable to DNA damage.

On the basis of a two hit hypothesis, we conclude that the first hit is the modification of the *FUS* gene leading to a cytoplasmic mislocalization of mFUS in MN depending upon the severity of the mutation that correlates with the onset of the disease (early onset, higher degree of FUS mislocalization). The second hit is an external factor such as DNA damage or stress. In young control cells, FUS was mislocalized only after the induction of DNA damage, in mFUS large FUS positive inclusions were seen. A strong FUS mislocalization was also detectable in older mFUS MNs. We hypothesize that this is due to neuronal maturation/aging and a later event during ALS pathology. In hiPSC derived MNs only a few larger FUS^+^ inclusions were detected giving rise to the supposition that possibly a third hit is needed to increase the size and toxicity of FUS inclusions that finally induce neuronal degeneration.

## Author Contributions

TMB, MD, and JH designed and outlined the study. MD and JH carried out all experiments and analyzed the images. SL, SP, A-KL, A-KH, MK, and AH reprogrammed and characterized the hiPSC cell lines. GB analyzed the karyotypes. JB and GS performed and analyzed the results from the comet assay. JD and CJ contributed to the image analysis and created a program to analyze protein co-localization. TMB, MD, and JH jointly wrote the manuscript. ACL, AMH, JHW, and AH provided the keratinocytes and the fibroblasts and the participants clinical information.

## Conflict of Interest Statement

The authors declare that the research was conducted in the absence of any commercial or financial relationships that could be construed as a potential conflict of interest.

## Footnotes

1 www.imageJ.nih.gov

## Supplementary Material

FIGURE S1 **Pluripotency characteristics of the newly generated FUS3 line Asp502ThrfS^∗^27. (A–D)** The newly generated iPSC cell line Asp502ThrfS^∗^27 was tested for specific markers to ensure pluripotency. **(A)** The hiPS cell line expressed the nuclear factors (all green) octamer- binding transcription factor 4 (OCT4), sex determining region Y-box 2 (SOX2) and nanog homeobox (NANOG) and the characteristic surface markers (all red) stage-specific embryonic antigen 4 (SSEA-4), tumor-related antigen (TRA) 1-60 and TRA1-81. **(B)** Undifferentiated iPSCs showed high endogenous mRNA levels for *OCT4*, *SOX2*, and *NANOG*, whereas *KLF4* levels are low compared to keratinocytes. Expression levels are shown relative to the housekeeping gene *HMBS*. **(C)** The iPSC colonies were able to differentiate into all three germ layers. Immunostainings showed cells positive for ectodermal (tubulin-β-II), mesodermal (actinin) and endodermal (AFP) protein markers. Scale bars: 50 μm. (**D**) Cell line Asp502ThrfS^∗^27 represented a normal male karyotype (46, XY) after the reprogramming process. **(E)** The localization of FUS in all control (CNTL1-3) and ALS-FUS patient-derived hiPSCs lines (R521C, R495QfsX527, Asp502ThrfS^∗^27) was predominantly limited to the nucleus. In some cells, low amounts of cytoplasmic FUS could be observed. hiPSC line FUS3, expressing mFUS Asp502ThrfS^∗^27, displayed higher amounts of cytoplasmic FUS compared to the other cell lines. Scale bars: 10 μm.

FIGURE S2 **The isogenic control R495QfsX527^c.1483insC^ line shows no cytoplasmic FUS mislocalization. (A)** Illustration, showing the DNA and amino acid (AA) sequence of the patient cell line R495QfsX527 and the corrected cell line R495QfsX527^c.1483insC^, in which a C-nucleotide at position 1483 of exon 14 was inserted via CRISPR technology. The novel 1 bp deletion c.1483delC (highlighted in red) leads to a frameshift (gray arrow) and the translation of new AAs before the STOP codon at AA-position 540. **(B)** Alignment of the sequencing results of genomic CNTL and R495QfsX527^c.1483insC^ revealed no further alteration within the fragment besides the C-insertion (shown in red) and the two PAM sequences (both green). **(C)** The “rescued” cell line R495QfsX527^c.1483insC^ was tested for specific pluripotency markers. The hiPS cell line expressed the nuclear factors (all green) SOX2, OCT4 and NANOG and the characteristic surface markers (all red) SSEA-4, TRA1-60 and TRA1-81. R495QfsX527^c.1483insC^ showed high endogenous mRNA levels for *SOX2, OCT4* and *NANOG*, whereas *KLF4* levels were low. Expression levels are shown relative to the housekeeping gene *HMBS.* **(D)** The localization of FUS in R495QfsX527 expressing mFUS and R495QfsX527^c.1483insC^expressing the corrected protein was predominantly limited to the nucleus in hiPS cells. In both cell lines, low amounts of cytoplasmic FUS could be observed in only some cells. **(E)** In contrast to motoneurons expressing R495QfsX527, in which cytoplasmic FUS and FUS^+^ granules along the neurites were detected, the corrected version R495QfsX527^c.1483insC^showed predominantly nuclear FUS with only small amount of cytoplasmic FUS. **(F)** Quantification of γH2A.X^+^ cells before and 24 h after irradiation in hiPSCs and mature motoneurons. Results are displayed relative to untreated repaired control line. After irradiation, the R495QfsX527 as well as R495QfsX527^c.1483insC^ cell line showed an increased number of γH2A.X^+^ cells compared to non-irradiated and even differences amongst cell lines were detectable before and after irradiation in hiPS cells (unpaired *t*-test, comparison of cell lines was performed by one way ANOVA with Bonferroni *post hoc* test) ^∗^*p* ≤ 0.05, ^∗∗^*p* ≤ 0.01, ^∗∗∗^*p* ≤ 0.001 All scale bars: 10 μm.

FIGURE S3 **hiPSC-derived motoneurons express specific motoneuronal markers and develop a dense neuronal network. (A)** Twenty-one days old motoneurons were immunostained for motoneuronal markers HB9, Islet-1 (ISL-1) and choline acetyltransferase (ChAT) (all in green) to demonstrate motoneuron differentiation. At this stage, differentiated hiPSCs expressed the subtype specific markers HB9 and ISL-1, transcription factors, localized in the nucleus of the cells. Additionally, motoneurons were positive for ChAT. **(B,C)** Developing motoneurons were tested for the neuronal marker Tubulin beta-III (TUJ1) (blue) and the axonal marker neurofilament heavy chain (NF-H) (magenta). On day 21 of motoneuronal differentiation all control and mFUS-derived cell lines developed a dense neuronal network, positive for the early motoneuronal marker TUJ1 **(B)**. From day 42 onwards the neuronal network becomes more complex and cells expressed the subtype specific axonal marker NF-H **(C)**. Scale bars:10 μm.

## Abbreviations

AA: amino acid
AFP: alpha fetoprotein
ALS: amyotrophic lateral sclerosis
ChAT: choline acetyltransferase
CRISPR: clustered regularly interspaced short palindromic repeats
DAPI: 4′,6-diamidino-2-phenylindole
DDR: DNA-damage response
EB: embryoid body
FGF: fibroblast grow factor
FTLD: frontotemporal lobar degeneration
FUS: fused in sarcoma
HDAC1: histone deacetylase-1
hiPSC: human induced pluripotent stem cells
HB9: homeobox gen-9
iPSC: induced pluripotent stem cells
KLF4: Kruppel-like factor 4
MAP-2: microtubule associated protein-2
MEFs: mouse embryonic fibroblasts
mFUS: mutated FUS
MN: motoneuron
NANOG: nanog homeobox
NF-H: neurofilament heavy chain
NLS: nuclear localization signal
OCT4: octamer-binding transcription factor 4
SEM: standard error of the mean
SG: stress granule
SOD1: super oxide dismutase-1
SOX2: sex determining region Y-box 2
SSEA4: stage-specific embryonic antigen
TDP-43: TAR DNA-binding protein
TRA-1: tumor-related antigen
WT FUS: wild type FUS.
