# Supplementary figures and images for: FUS Mislocalization and Vulnerability to DNA Damage in ALS Patients Derived hiPSCs and Aging Motoneurons
Source: Front Cell Neurosci. 2016 Dec 26;10:290. doi: 10.3389/fncel.2016.00290 (PMC5183648; doi:10.3389/fncel.2016.00290)

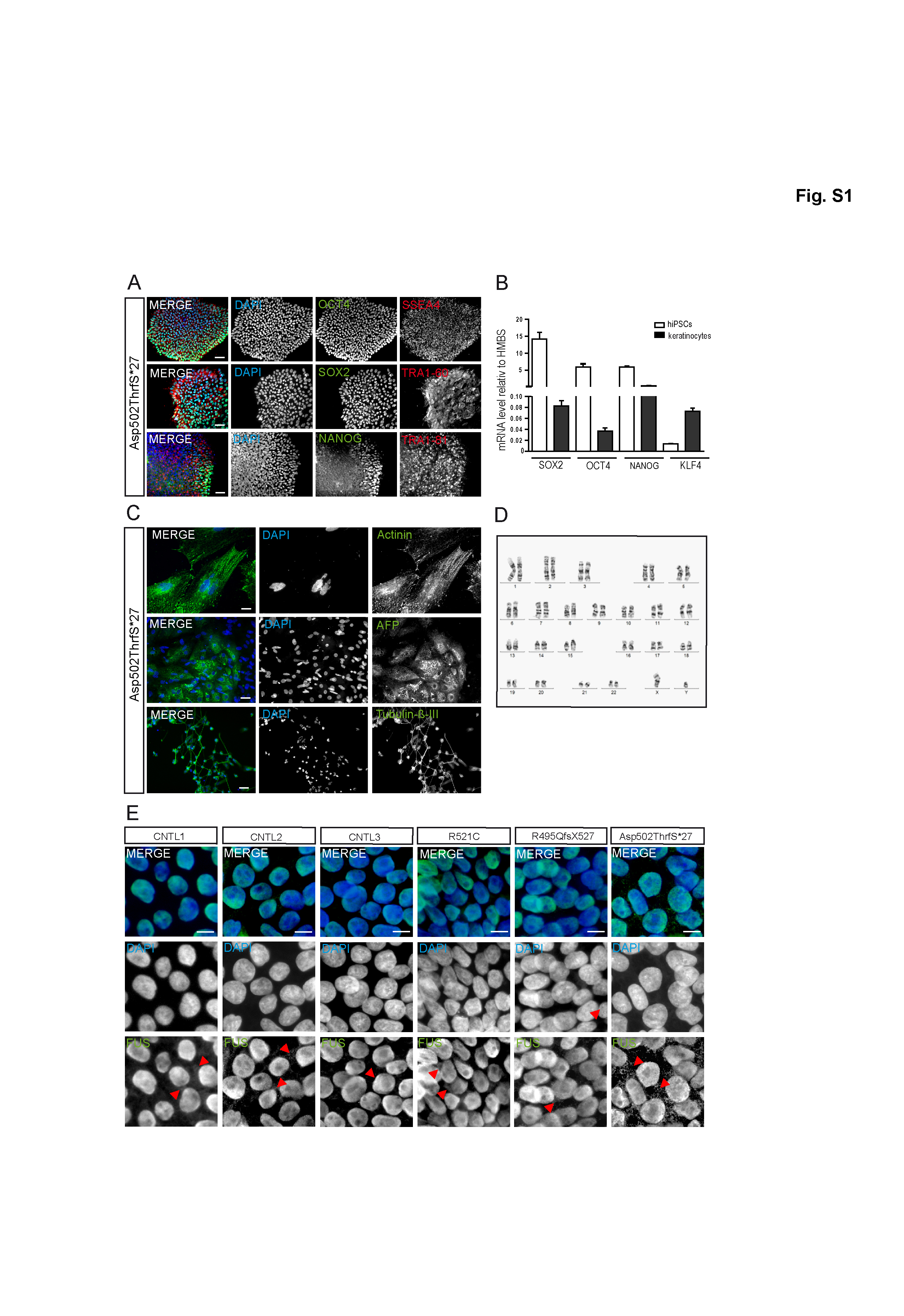

Supplement: FIGURE S1 — Pluripotency characteristics of the newly generated FUS3 line Asp502ThrfS∗27. (A–D) The newly generated iPSC cell line Asp502ThrfS∗27 was tested for specific markers to ensure pluripotency. (A) The hiPS cell line expressed the nuclear factors (all green) octamer- binding transcription factor 4 (OCT4), sex determining region Y-box 2 (SOX2) and nanog homeobox (NANOG) and the characteristic surface markers (all red) stage-specific embryonic antigen 4 (SSEA-4), tumor-related antigen (TRA) 1-60 and TRA1-81. (B) Undifferentiated iPSCs showed high endogenous mRNA levels for OCT4, SOX2, and NANOG, whereas KLF4 levels are low compared to keratinocytes. Expression levels are shown relative to the housekeeping gene HMBS. (C) The iPSC colonies were able to differentiate into all three germ layers. Immunostainings showed cells positive for ectodermal (tubulin-β-II), mesodermal (actinin) and endodermal (AFP) protein markers. Scale bars: 50 μm. (D) Cell line Asp502ThrfS∗27 represented a normal male karyotype (46, XY) after the reprogramming process. (E) The localization of FUS in all control (CNTL1-3) and ALS-FUS patient-derived hiPSCs lines (R521C, R495QfsX527, Asp502ThrfS∗27) was predominantly limited to the nucleus. In some cells, low amounts of cytoplasmic FUS could be observed. hiPSC line FUS3, expressing mFUS Asp502ThrfS∗27, displayed higher amounts of cytoplasmic FUS compared to the other cell lines. Scale bars: 10 μm. [file Image_1.JPEG]

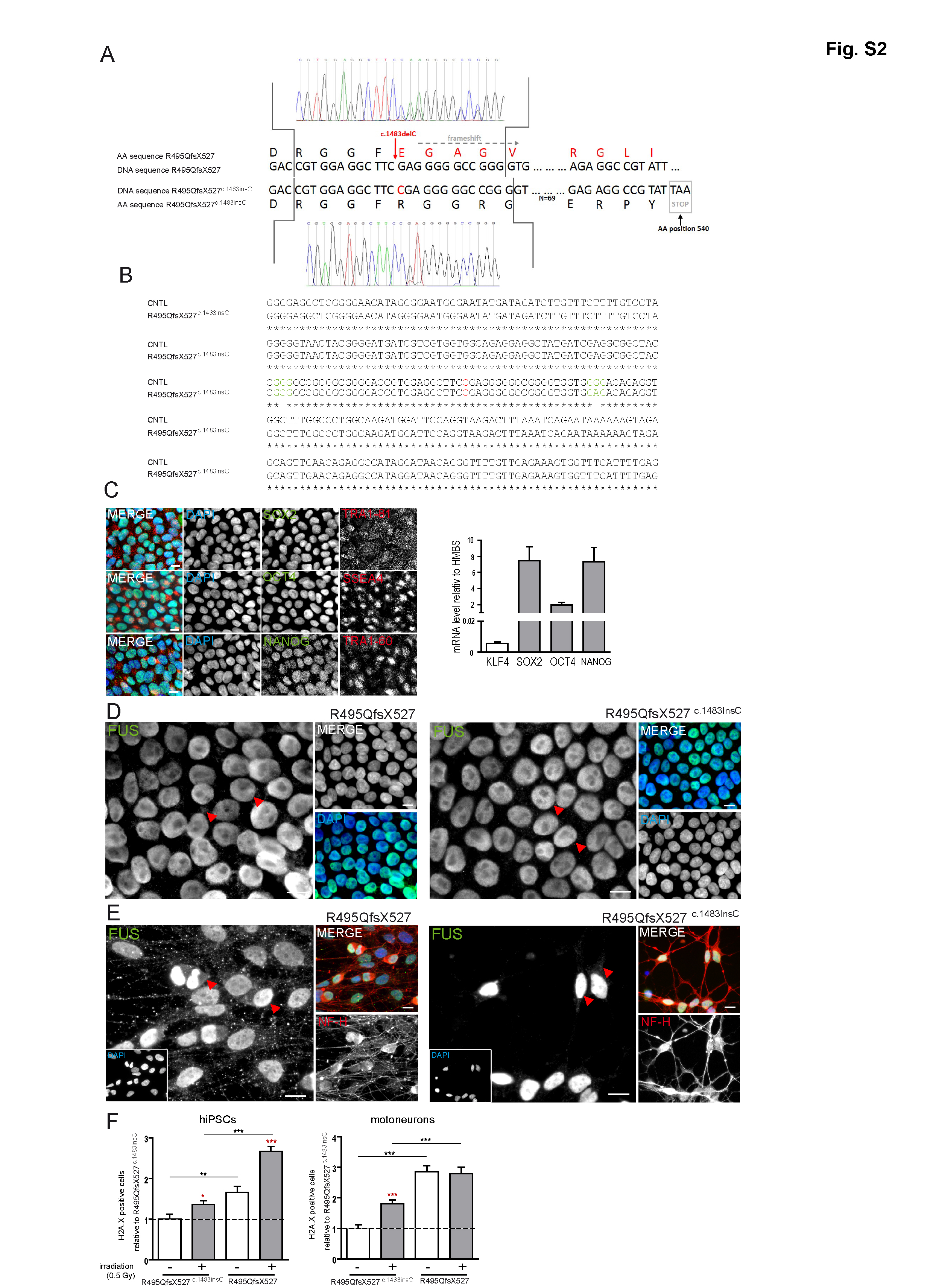

Supplement: FIGURE S2 — The isogenic control R495QfsX527c.1483insC line shows no cytoplasmic FUS mislocalization. (A) Illustration, showing the DNA and amino acid (AA) sequence of the patient cell line R495QfsX527 and the corrected cell line R495QfsX527c.1483insC, in which a C-nucleotide at position 1483 of exon 14 was inserted via CRISPR technology. The novel 1 bp deletion c.1483delC (highlighted in red) leads to a frameshift (gray arrow) and the translation of new AAs before the STOP codon at AA-position 540. (B) Alignment of the sequencing results of genomic CNTL and R495QfsX527c.1483insC revealed no further alteration within the fragment besides the C-insertion (shown in red) and the two PAM sequences (both green). (C) The “rescued” cell line R495QfsX527c.1483insC was tested for specific pluripotency markers. The hiPS cell line expressed the nuclear factors (all green) SOX2, OCT4 and NANOG and the characteristic surface markers (all red) SSEA-4, TRA1-60 and TRA1-81. R495QfsX527c.1483insC showed high endogenous mRNA levels for SOX2, OCT4 and NANOG, whereas KLF4 levels were low. Expression levels are shown relative to the housekeeping gene HMBS. (D) The localization of FUS in R495QfsX527 expressing mFUS and R495QfsX527c.1483insCexpressing the corrected protein was predominantly limited to the nucleus in hiPS cells. In both cell lines, low amounts of cytoplasmic FUS could be observed in only some cells. (E) In contrast to motoneurons expressing R495QfsX527, in which cytoplasmic FUS and FUS+ granules along the neurites were detected, the corrected version R495QfsX527c.1483insCshowed predominantly nuclear FUS with only small amount of cytoplasmic FUS. (F) Quantification of γH2A.X+ cells before and 24 h after irradiation in hiPSCs and mature motoneurons. Results are displayed relative to untreated repaired control line. After irradiation, the R495QfsX527 as well as R495QfsX527c.1483insC cell line showed an increased number of γH2A.X+ cells compared to non-irradiated and even differences amon [file Image_2.JPEG]

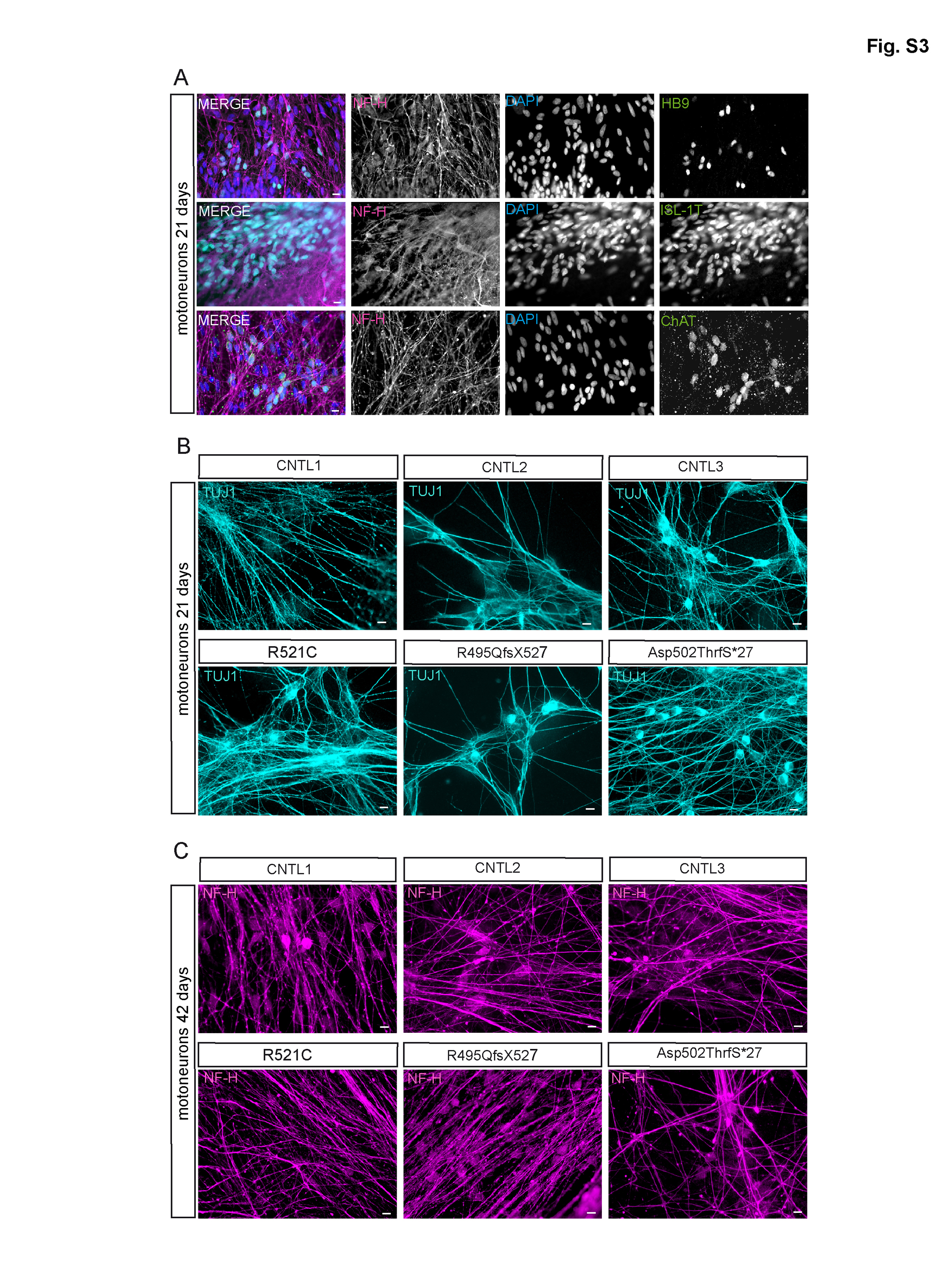

Supplement: FIGURE S3 — hiPSC-derived motoneurons express specific motoneuronal markers and develop a dense neuronal network. (A) Twenty-one days old motoneurons were immunostained for motoneuronal markers HB9, Islet-1 (ISL-1) and choline acetyltransferase (ChAT) (all in green) to demonstrate motoneuron differentiation. At this stage, differentiated hiPSCs expressed the subtype specific markers HB9 and ISL-1, transcription factors, localized in the nucleus of the cells. Additionally, motoneurons were positive for ChAT. (B,C) Developing motoneurons were tested for the neuronal marker Tubulin beta-III (TUJ1) (blue) and the axonal marker neurofilament heavy chain (NF-H) (magenta). On day 21 of motoneuronal differentiation all control and mFUS-derived cell lines developed a dense neuronal network, positive for the early motoneuronal marker TUJ1 (B). From day 42 onwards the neuronal network becomes more complex and cells expressed the subtype specific axonal marker NF-H (C). Scale bars:10 μm. [file Image_3.JPEG]
